# Genomic evidence for homoploid hybrid speciation in a marine mammal apex predator

**DOI:** 10.1126/sciadv.adf6601

**Published:** 2023-05-03

**Authors:** Fernando Lopes, Larissa R. Oliveira, Yago Beux, Amanda Kessler, Susana Cárdenas-Alayza, Patricia Majluf, Diego Páez-Rosas, Jaime Chaves, Enrique Crespo, Robert L. Brownell, Alastair M. M. Baylis, Maritza Sepúlveda, Valentina Franco-Trecu, Carolina Loch, Bruce C. Robertson, Claire R. Peart, Jochen B. W. Wolf, Sandro L. Bonatto

**Affiliations:** ^1^ Escola de Ciências da Saúde e da Vida, Pontifícia Universidade Católica do Rio Grande do Sul, PUCRS, Porto Alegre, Brazil.; ^2^ Laboratório de Ecologia de Mamíferos, Universidade do Vale do Rio dos Sinos, São Leopoldo, Brazil.; ^3^ Finnish Museum of Natural History, University of Helsinki, Helsinki, Finland.; ^4^ Grupo de Estudos de Mamíferos Aquáticos do Rio Grande do Sul (GEMARS), Torres, Brazil.; ^5^ Centro para la Sostenibilidad Ambiental, Universidad Peruana Cayetano Heredia, Lima, Peru.; ^6^ Departamento de Ciencias Biológicas y Fisiológicas, Facultad de Ciencias y Filosofía, Universidad Peruana Cayetano Heredia, Lima, Peru.; ^7^ Colegio de Ciencias Biológicas y Ambientales, COCIBA, Universidad San Francisco de Quito, Quito, Ecuador.; ^8^ Dirección del Parque Nacional Galápagos, Oficina Técnica San Cristobal, Islas Galápagos, Ecuador.; ^9^ Galapagos Science Center, Puerto Baquerizo Moreno, Ecuador.; ^10^ Department of Biology, San Francisco State University, 1800 Holloway Ave, San Francisco, CA, USA.; ^11^ Laboratório de Mamíferos Marinos, CESIMAR - CCT CENPAT, CONICET, Puerto Madryn, Argentina.; ^12^ Southwest Fisheries Science Center, NOAA Fisheries, La Jolla, CA, USA.; ^13^ South Atlantic Environmental Research Institute, Stanley, Falkland Islands.; ^14^ Centro de Investigación y Gestión de Recursos Naturales (CIGREN), Facultad de Ciencias, Universidad de Valparaíso, Valparaíso, Chile.; ^15^ Departamento de Ecología y Evolución, Facultad de Ciencias, Universidad de la República, Montevideo, Uruguay.; ^16^ Sir John Walsh Research Institute, Faculty of Dentistry, University of Otago, Dunedin, New Zealand.; ^17^ Department of Zoology, University of Otago, Dunedin, New Zealand.; ^18^ Division of Evolutionary Biology, LMU Munich, München, Germany.

## Abstract

Hybridization is widespread and constitutes an important source of genetic variability and evolution. In animals, its role in generating novel and independent lineages (hybrid speciation) has been strongly debated, with only a few cases supported by genomic data. The South American fur seal (SAfs) *Arctocephalus australis* is a marine apex predator of Pacific and Atlantic waters, with a disjunct set of populations in Peru and Northern Chile [Peruvian fur seal (Pfs)] with controversial taxonomic status. We demonstrate, using complete genome and reduced representation sequencing, that the Pfs is a genetically distinct species with an admixed genome that originated from hybridization between the SAfs and the Galapagos fur seal (*Arctocephalus galapagoensis*) ~400,000 years ago. Our results strongly support the origin of Pfs by homoploid hybrid speciation over alternative introgression scenarios. This study highlights the role of hybridization in promoting species-level biodiversity in large vertebrates.

## Teaser

Genomic analyses reveal the Peruvian fur seal originated from hybrid speciation between the Galapagos and South American fur seals.

## INTRODUCTION

Homoploid hybrid speciation (HHS) refers to the stable establishment of hybrid populations attaining evolutionary independence from their parental donor species without change in ploidy (*1*). The composition of parental ancestry components can, in principle, range from the transfer of single genes to the dominance of alleles from the more abundant species to proportions approaching parity (*2*–*5*). Recombination of parental material results in hitherto unproven haplotypic combinations and allows exploration of novel phenotypes and environmental niche space that was inaccessible to the parents (*6*–*8*). Combined with geographical isolation following founder events, hybrid genomes can stabilize before being swamped by homogenizing gene flow with the generally more abundant parental species (*6*–*9*).

The conditions that must be satisfied to accept a case as HHS are debated, ranging from mere identification of a new hybrid lineage that is stable and reproductively isolated from the parental species (*10*) to more restrictive definitions requiring evidence that the hybridization was the leading cause for the reproductive isolation (RI) (*11*). However, the last criterion is disputed on both empirical and conceptual grounds (*10*). Determining whether the RI of the new lineage was directly derived from the hybridization event may be very difficult in ancient hybridizations (*9*, *12*), and artificial crosses allowing inference of hybrid fitness (*11*) apply to only a subset of organisms. Moreover, the mode and strength of RI deemed relevant for speciation differ by species concept and taxon (*13*), blurring the criteria that could be applied across the tree of life. Experimental evidence for hybrid speciation remains accordingly scarce (*9*, *14*, *15*). Genome-wide molecular evidence recognizes a broader range of candidate cases in plants and small-body animals (*9*, *10*, *16*). However, even considering the less strict definitions, cases in which HHS may be considered a credible hypothesis given convincing evidence (e.g., genomic data) seem extremely rare in large vertebrates and, to our knowledge, have not been reported in mammals (*17*).

**Fig. 1.**
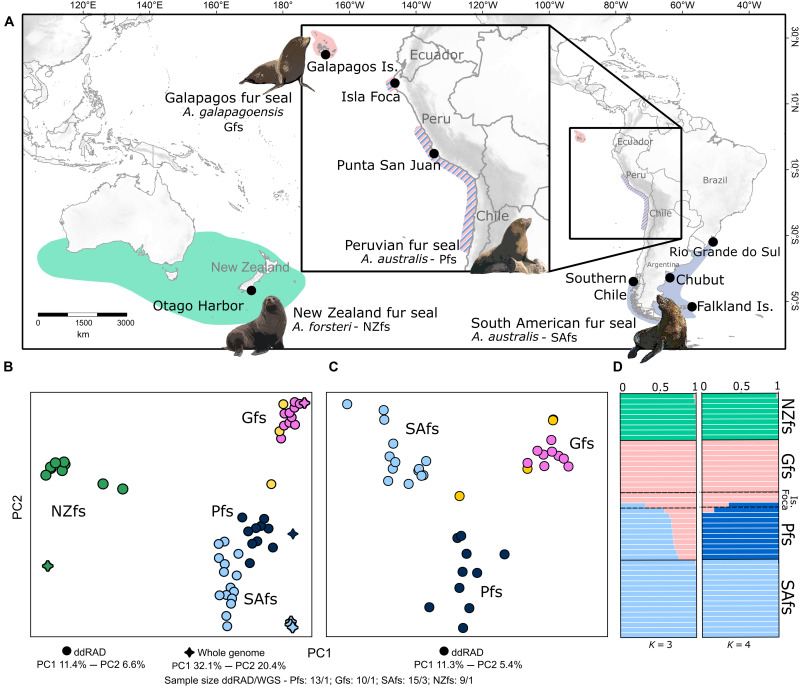
Sampling setup and population structuring of the focal taxa. (**A**) Distribution of fur seal species and localities assessed in this study. From the left to the right: New Zealand fur seal (NZfs), *A. forsteri*, green; Galapagos fur seal (Gfs), *A. galapagoensis*, pink; Peruvian fur seal (Pfs) *A. australis*, hatched blue and pink lines; and South American fur seal (SAfs), *A. australis*, light blue. (**B**) Principal components analysis (PCA) of superimposed whole-genome resequencing (stars) and double digest restriction site–associated DNA sequencing (ddRAD-seq) (circles) data. (**C**) PCA of ddRAD-seq data with only the three focal taxa. WGS, whole-genome shotgun. (**D**) Admixture plots based on ddRAD-seq data with *K* = 3 and 4. Samples from Isla Foca are indicated in yellow in (B) and (C) and are specified in (D).

Here, we present results of whole-genome and reduced representation sequencing from three species and subpopulations of fur seals, large marine apex carnivores, previously found to have a complex evolutionary history with evidence of past hybridization (Fig. 1A) (*18*). The Galapagos fur seal (Gfs; *Arctocephalus galapagoensis*) is endemic to the Galapagos Archipelago (*19*), and the New Zealand fur seal (NZfs; *Arctocephalus forsteri*) is distributed in New Zealand and Southern Australia (Fig. 1A) (*20*). The South American fur seal (SAfs; *Arctocephalus australis*) has two disjunct distribution ranges: Most rookeries occur continuously from the southern Atlantic coast of southern Brazil to the southern Pacific coast of Chile. The second range begins 2000 km northward, runs from the north-central Chilean coast to the central Peruvian coast, and is concentrated in relatively few reproductive colonies, the largest in Punta San Juan (PSJ), central-southern Peru (Fig. 1A) (*21*). Several studies have found that individuals from the latter populations have distinct morphological, molecular (*22*–*24*), and biological features (*25*). These have motivated authors to regard the Peruvian fur seal (Pfs) as a biological entity of its own: as an Evolutionarily Significant Unit (*23*) or a subspecies of *A. australis* (*26*, *27*). A small and isolated rookery of putative Pfs is also present on Isla Foca, Peru ~1000 km north of the main distribution (*28*) and within the area of convergence of the Humboldt Current and the Equatorial Current, a region with both temperate and cold-water marine species. We characterized the genomic diversity, population relationships, and evolutionary history of the group, which allowed us to delimit species, estimate speciation scenarios, and assess signals of genomic introgression. These results establish that the Peruvian fur seal is a new species that originated from HHS between the Galapagos fur seal and the South American fur seal.

## RESULTS

### Sequencing, SNP filtering information, and ploidy characterization

In addition to previously sequenced genomes of the NZfs, Gfs, and SAfs (from Argentina) (*18*), we added two new genomes of the latter from Chile and Falklands/Malvinas (hereafter Falklands) and one Pfs (Fig. 1A). Our Peruvian sample came from the PSJ colony, the largest on the Peruvian/Northern Chilean coast (33 to 42% of the total amount of individuals), which is responsible for producing the largest number of pups per reproductive season (*28*). The previous studies that observed the distinctiveness of the Pfs used primarily individuals from PSJ (see Introduction). The average coverage, including the published genome of the Antarctic fur seal (Afs; *Arctocephalus gazella*) used as an outgroup, was 24.6× (±6.2) (table S1). The filtered whole-genome panel consisted of ~13.3 million single-nucleotide polymorphisms (SNPs) distributed in 3892 scaffolds. We also generated a double digest Restriction-site Associated DNA sequencing (ddRAD-seq) SNP dataset resulting in a filtered dataset of 47 individuals from all species and populations with a total of 3198 segregating SNPs distributed in 6168 scaffolds (Fig. 1, figs. S1 and S2, and table S2).

By definition, an essential requirement of the HHS model is that the new hybrid lineage originated without change in chromosome number; specifically, it should not be polyploid (*9*). Polyploidy is considered extremely unlikely, if not impossible, in mammals, and no viable polyploid mammal has been found so far (*29*). Nonetheless, we used the whole-genome shotgun (WGS) data to estimate the ploidy (both polyploidy and aneuploidy) of our focal species. The results support that the Gfs, Pfs, and SAfs genomes are diploids, as was already found for the SAfs (*30*), and that none is an aneuploid (fig. S3).

### Population structure and genetic differentiation

We first explored genetic differentiation between individuals and between species using principal components analysis (PCA) with both the whole-genome (13,316,418 SNPs) and ddRAD-seq datasets. In both cases, species were well separated, and individuals of the same species clustered together, including the SAfs of different populations (Brazil, Argentina, Falklands, and Chile) (Fig. 1, B and C, and fig. S4). However, Pfs assumed an intermediate position between the SAfs and the Gfs. Considering the Pfs and its putative parents in isolation, a “V” pattern characteristic of hybrid origin (*31*) suggested a near-equal contribution of Gfs and SAfs ancestry (equidistance on PC 1), followed by an independent history of Pfs (shift along PC 2) (Fig. 1C).

Genetic differentiation between populations and species estimated with *F*_ST_ (ddRAD-seq data) was concordant with the above results, presenting a low to moderate population differentiation (0.03 to 0.09) between the four South American populations (table S3). *F*_ST_ between these localities and the Pfs was higher, ranging from 0.13 (Pfs-SAfs Chile) to 0.18 (Pfs-SAfs Falklands). *F*_ST_ between recognized species ranged from 0.20 (SAfs Chile-NZfs) to 0.40 (Gfs-NZfs) (table S3).

Admixture analysis with ddRAD-seq data agreed with PCA results, supporting three genetic clusters (*K* = 3 had the lowest cross-validation error; fig. S5), with individuals of each species appearing as a genetically homogeneous group (Fig. 1D). However, all Pfs from the mainland (PSJ colony) were admixed, sharing ancestry components from the SAfs and Gfs, with the dominance of SAfs ancestry. With *K* = 4 to 6 clusters (Fig. 1D and fig. S5), the Pfs presented a distinct genetic component. Except for one individual that showed a small proportion (~12%) of Galapagos ancestry, all mainland individuals of the Pfs (from PSJ) were noticeably homogeneous in their ancestry. All individuals from PSJ had mtDNA haplotypes representative of the Pfs (table S4) (*30*).

Two of the three Peruvian individuals sampled in the isolated rookery in Isla Foca (~1000 km distant north from PSJ on the northern coast of Peru) grouped with the Galapagos individuals in the PCA (Fig. 1, B and C). They also contained only the Galapagos genetic component in the admixture results (Fig. 1D) and mitochondrial DNA (mtDNA) signatures of the Gfs (table S4). The third individual from Isla Foca was positioned between the Gfs and the Peruvian samples, suggesting an F2 backcrossed hybrid between Pfs and Gfs, with 75% of its genome from the former and 25% from the latter (Fig. 1). This individual had the mtDNA signature of the Pfs (table S4).

### Phylogenetic relationships

**Fig. 2.**
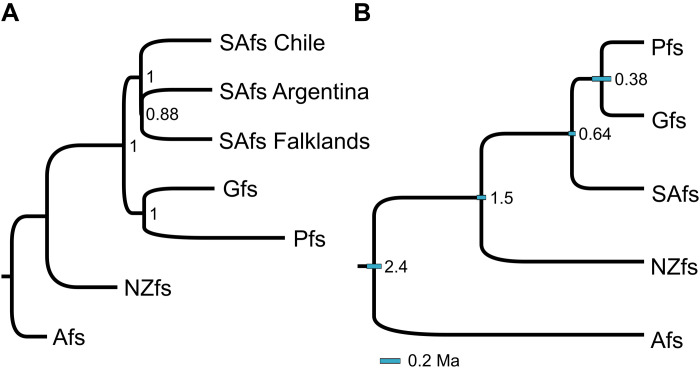
Species trees and divergence time among fur seals species. (**A**) Species tree estimated with ASTRAL-III based on 7810 independent gene trees. Numbers are node support. (**B**) StarBEAST2 species tree with point estimates of divergence times based on 300 independent loci of 50 kb. Node numbers are the divergence times (million years ago). Blue bars represent the 95% confidence interval. The bar at the bottom of the phylogeny is a scale bar for the confidence interval. All nodes with support = 1. NZfs, *A. forsteri*; Gfs, *A. galapagoensis*; Pfs, *A. australis*; SAfs, *A. australis*; and the outgroup, the Afs, *A. gazella*.

We next estimated the phylogenetic relationships between the individual genomes using a multispecies coalescent species tree approach with ASTRAL-III (Fig. 2A). The genomes from the three South American populations grouped with full support in an almost polytomous clade, with the two genomes from the Atlantic (Argentina and Falklands) grouping with partial support. Unexpectedly, the Pfs was sister to the Gfs, with full support and presented a more extended branch. The NZfs was sister to SAfs and Gfs + Pfs clades. Next, we used the StarBEAST2 species tree approach to estimate relationships and divergence times based on 300 genomic regions. Given the results described above, we kept the Peruvian sample as an independent taxon (Fig. 2B). Again, the Peruvian and Gfs were sister species with relatively recent divergence (~0.4 Ma ago). This clade diverged from the rest of the SAfs ~0.65 Ma and the NZfs diverged around 1.5 Ma. This species tree was identical, and divergence times were very similar to our previous study, which only used the individual from Argentina to represent the SAfs (*18*). Contrary to the nuclear genome, the phylogenetic tree of the mitochondrial genomes grouped the Peruvian mtDNA with the SAfs, although being the most divergent lineage (fig. S6). The SAfs sample from Argentina was sister to the group from Chile and Falklands, corroborating previous findings (*18*, *32*, *33*).

**Fig. 3.**
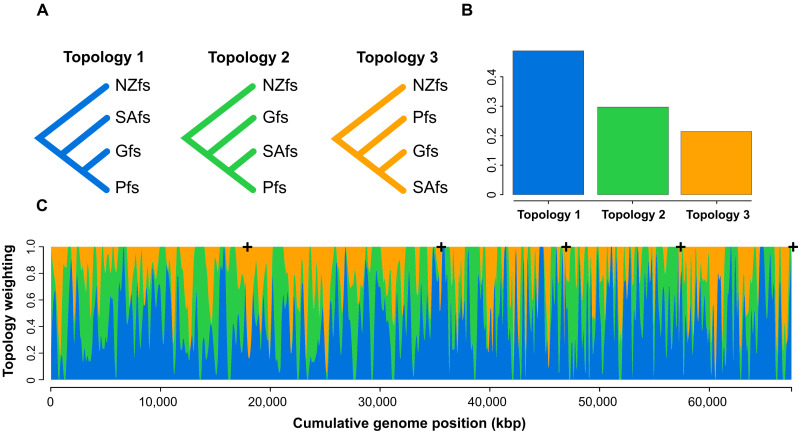
Topology weighting across the genome for the origin of the Pfs. (**A**) The main three alternative topologies for the position of the Pfs and (**B**) their relative weightings across the whole genomes. (**C**) Smoothed weights across the five largest scaffolds (delimited by the plus signals).

Given the discordant phylogenetic position of the Pfs in different analyses, we next quantified the genomic frequency of the three different topologies considering Pfs, Gfs, and one of the SAfs genomes (Fig. 3A). We split each of the 724 largest scaffolds (>1 Mb), which comprise about 1.9 Gb, into contiguous windows of 50 kb. Maximum likelihood trees were estimated for each of the 38,823 genomic windows. In 51% of the trees, the Pfs grouped with Gfs, 29% grouped with the SAfs, and 20% Gfs and SAfs were grouped (Fig. 3B). We next used the Twisst analysis to better understand the distribution of these topologies along the largest scaffolds. The three topologies were distributed relatively regularly along the larger scaffolds (Fig. 3C and figs. S7 and S8). This high level of discordance may be the consequence of past hybridization, and its smooth distribution suggests that it occurred many generations ago, as recombination at each generation continuously breaks down long tracks of the parental chromosomes (*5*). However, because incomplete lineage sorting (ILS) can result in a similar pattern, we next used several methods to test for evidence of past hybridization events.

### Genomic introgression among populations and species

We applied the *f*_3_ and *f*_4_-statistics to investigate signals of admixture between populations by splitting up the genome into 1331 blocks of 10,000 SNPs each. The *f*_3_-statistic explicitly tests whether a taxon of interest *C* results from admixture between two others (*A* and *B*): A significantly negative *f*_3_-statistic supports the admixture hypothesis, while a positive value is not informative (*34*). The *f*_3_ analysis showed strong signals that Pfs is admixed between the SAfs and the Gfs (table S5); signals were similar regardless of the choice of SAfs localities (Argentina, Falklands, and Chile). Although substantially weaker, we also detected an introgression signal between the NZfs and Gfs. The *f*_4_-statistics use four-taxa topologies in the form (*A*,*B*;*C*,*D*), and when *A* is an outgroup, it allows testing past introgression between *B* and either *C* or *D*. A significantly positive *f*_4_ implies gene flow between *B* and *D*, and a significantly negative *f*_4_ implies gene flow between *B* and *C*. Here, *A* is either NZfs or Afs, *B* is SAfs or Gfs, and we fixed Pfs as *D* to ease visualization (this only affects the sign). Because the phylogenetic position of the Pfs as a sister to the Gfs or the SAfs was unclear, we tested both topologies. We found highly significant signals of gene flow between Pfs and both Gfs and SAfs (Table 1 and fig. S9). We also found a weak introgression signal between NZfs and SAfs (*f*_4_ < 0.003). These results strongly supported that the Pfs was involved in past gene flow with both the Gfs and the SAfs, although these statistics cannot discern the direction of the introgressions nor distinguish between a scenario of continuous gene flow and HHS.

**Table 1.** *f*_4_-statistics test of introgression between the Pfs, SAfs, and Gfs. For *f*_4_ (*A*,*B*;*C*,*D*), a significantly positive *z* score implies gene flow between *B* and *D*. Only four-taxa topologies that include the Peruvian fur seal (Pfs) and its putative parental species, the Galapagos (Gfs) and the South American [SAfs Argentina (AR), Falkland Islands (FK), and Chile (CH)] fur seals, were shown here. New Zealand (NZfs) and Antarctic fur seals (Afs) were used as outgroups. All results are highly significant (*z* scores: > 3).

(*A*	*B*)	(*C*	*D*)	*f*_4_-statistics	*z* score
NZfs	Gfs	SAfs FK	Pfs	0.02	65.76
NZfs	Gfs	SAfs CH	Pfs	0.019	65.16
NZfs	Gfs	SAfs AR	Pfs	0.019	63.83
Afs	Gfs	SAfs FK	Pfs	0.015	58.26
Afs	Gfs	SAfs CH	Pfs	0.014	58.71
Afs	Gfs	SAfs AR	Pfs	0.014	57.3
NZfs	SAfs FK	Gfs	Pfs	0.007	32.1
NZfs	SAfs CH	Gfs	Pfs	0.008	34.14
NZfs	SAfs AR	Gfs	Pfs	0.007	34.12
Afs	SAfs FK	Gfs	Pfs	0.007	39.13
Afs	SAfs CH	Gfs	Pfs	0.007	38.56
Afs	SAfs AR	Gfs	Pfs	0.007	39.57

**Fig. 4.**
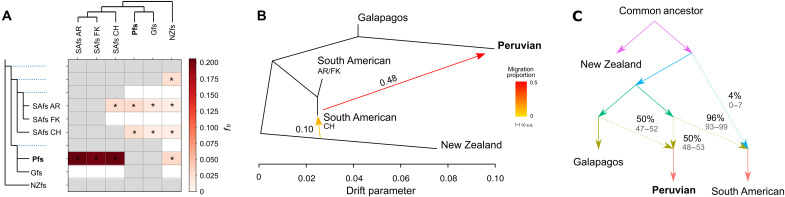
Peruvian fur seals are a hybrid species. (**A**) *f*-branch results. The values in the matrix refer to excess allele sharing (*f_b_* statistics) between the tree branches in the *y* axis and the species/populations on the *x* axis. Dotted lines in the phylogeny represent ancestral lineage, and nondotted lines represent extant lineages. The asterisks indicate significant results (*P* < 0.01). Gray cells are inestimable relationships. AR (Argentina), FK (Falklands), and CH (Chile). (**B**) TreeMix maximum likelihood phylogeny with the standard error (s.e.) and the direction of introgression (arrows) and (**C**) admixture graph supported the Pfs as a mixed genome between the Gfs and the SAfs. Dotted lines are the direction of introgression, and numbers are the percentage of introgression with their respective confidence intervals in gray. SAfs from AR and FK are combined as an Atlantic population (B). An introgression between the NZfs and the SAfs was also detected. NZfs is the basal taxon.

Next, to better understand the signals of introgression in a phylogenetic context, we used the *f*-branch metric to summarize the *f*_4_ ratio values (an estimation of the ancestry proportions in an admixed population). We used the three SAfs genomes from different localities and the Pfs and Gfs as sister species. We found strong evidence of introgression (*P* < 0.01) between the Pfs and SAfs regardless of population choice for the SAfs (Fig. 4A). The method also detected introgression between NZfs and SAfs, which would have occurred before the divergence between the populations and the latter. A minor significant signal was detected between Pfs and NZfs that may be the consequence of the previous introgression between SAfs (ancestral to Pfs) and NZfs. The horizontal lines of lower correlated signals from SAfs from Argentina and Chile are likely not introgression events but a consequence of their shared ancestry (*35*). The topology with the Pfs as sister to the SAfs produced similar evidence of introgression, but now between Pfs and Gfs (fig. S10). Overall, these results support the hypothesis that Pfs constitutes an admixed genome.

Last, we used Hybrid Detection (HyDe) analysis (*36*) to further investigate the admixture scenarios found above. HyDe is a method that tests whether a population may have arisen via hybridization and estimates the proportion of parental contributions using genomic data (*37*). We tested all possible hybridization hypotheses among Gfs, SAfs, and Pfs, using NZfs as an outgroup. We examined the three populations of SAfs (Argentina, Falklands, and Chile) individually and as a group. In all tests, we found high support for the Pfs as a hybrid between the Gfs and the SAfs. None of the other scenarios, SAfs or Gfs as a hybrid, were supported (Table 2). In this analysis, the shared ancestry of the Pfs was higher with the SAfs (γ = 0.72) than with the Gfs.

**Table 2.** HyDe analysis at population and individual levels. Analyses used 97,047 single-nucleotide polymorphisms (SNPs) spaced by at least 25 kb. γ = shared ancestry between the putative hybrid and P2. *P* values (<0.05) indicate significant evidence of hybridization and are shown in bold.

P1	Hybrid	P2	*z* score	*P* value	γ
Population level					
**Galapagos**	**Peruvian**	**South American**	**4.65**	**<0.00001**	**0.72**
Peruvian	South American	Galapagos	−7.16	1	–
Peruvian	Galapagos	South American	−2.86	1	–
Individual level					
Galapagos	**Peruvian**	**SAfs CH**	**4.89**	**<0.00001**	**0.7**
Galapagos	**Peruvian**	**SAfs FK**	**4.05**	**<0.00001**	**0.75**
Galapagos	**Peruvian**	**SAfs AR**	**5**	**<0.00001**	**0.7**

### Phylogenomic inferences with admixture

On the basis of the evidence of substantial genome-wide admixture in the group, we next estimated the evolutionary relationship among these populations using methods that allow introgression. We first used TreeMix to infer a maximum likelihood tree of the populations with a given number of events of introgression using all possible *f*_4_ values as estimated above. The Pfs grouped with the SAfs, closest to the population from Chile and then to Argentina and Falklands, although the latter two groups had minimal branch lengths (fig. S11). However, about 49% of the Peruvian genome originated from an introgression event from the Gfs. Given the lack of differentiation between Argentina and Falklands samples of the SAfs, we ran a second analysis combining them as an Atlantic population (Fig. 4B). Here, the Pfs grouped with the Gfs, but 48% of its genome was introgressed from the Chilean SAfs, again the latter and the Atlantic populations were almost indistinguishable. The Pfs had a more extended branch (drift parameter) than the other samples in both analyses, indicating inflated genetic diversity, as is expected in admixed populations (*38*, *39*). In both analyses, TreeMix also detected small introgression from the NZfs to the SAfs, estimated as 4 or 10% (Fig. 4B and fig. S11). The admixture graph (summarizing results of the *f*_3_-statistics) showed similar results (Fig. 4C). Additional analysis with the three SAfs populations evaluated separately found the same results, although it suggests that the NZfs introgression would have occurred in the Atlantic populations (fig. S12).

Because all the above methods to estimate introgression use as input SNPs datasets, we next applied approaches that use gene trees as input. The phylogenomic analysis with PhyloNet and 7810 independent gene trees recovered four networks in a 95% credible set of topologies; the best ranked represented 55.9% of all networks, followed by the others representing 22.1, 15.4, and 5.4%, respectively (fig. S13). In all four networks, the Pfs was found as a hybrid between Gfs and SAfs, with the genomic contribution of the Gfs being higher (~70%). A smaller (~6%) introgressive event from the SAfs to the NZfs was detected.

### Testing HHS against alternative introgression scenarios

**Fig. 5.**
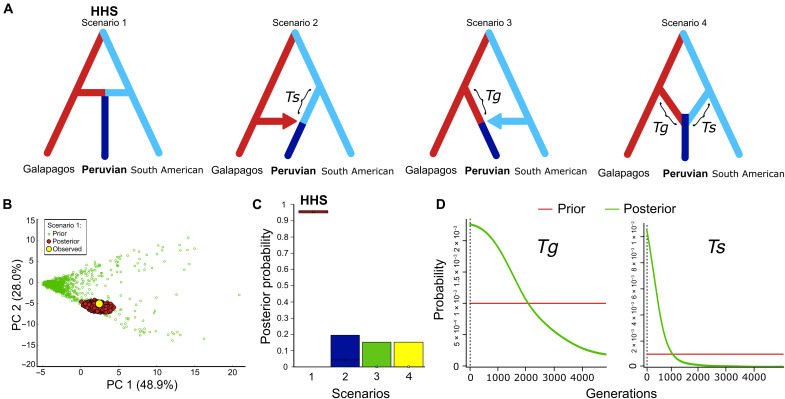
Test of demographic scenarios for the origin of the Pfs. (**A**) The HHS (scenario 1) and the three alternative introgression scenarios tested. *Ts* and *Tg* are the duration (in generations) of the intermediate subpopulation/s in the non–homoploid hybrid speciation (HHS) scenarios. (**B**) Model checking showing that the model fits the data well. (**C**) Posterior probability (point and 95% confidence interval) of each scenario (scenario choice) computed with logistic regression. (**D**) Posterior distributions of parameters *Ts* and *Tg* in scenarios 2 to 4 showing that they approached the minimum possible value of 0 (*x* axis).

Because our results highly supported a hybrid origin for the Pfs, we next used an approximate Bayesian computation approach to test whether the species originated by HHS or by alternative hybridization scenarios (Fig. 5A and fig. S14). Specifically, HHS implies a “nearly instantaneous” origin of a new lineage by hybridization between individuals from two parental species that remain otherwise unchanged, which requires the absence of previously differentiated subpopulations (of Gfs and SAfs) for the origin of the hybrid species and its simultaneous “divergence” from both parentals. The three alternative non-HHS scenarios were: Pfs originating from introgression (gene flow) of Gfs or SAfs into a previously isolated subpopulation of SAfs or Gfs (scenarios 2 and 3, respectively) and a hybrid origin of Pfs from the fusion of two previously isolated subpopulations from Gfs and SAfs (scenario 4). Scenario 4 may also be considered hybrid speciation, although it is biologically different from scenario 1. We found that the HHS scenario of instantaneous hybrid origin fits the data well (Fig. 5B) and is strongly supported (with posterior probability of >0.95) over the other non-HHS scenarios (Fig. 5C and fig. S15). For the non-HHS scenarios, we set a minimum of 100 generations for the duration of each subpopulation before the introgression or hybridization resulting in the Pfs (parameters *Tg* and *Ts*; Fig. 5D and fig. S14). This minimum time is necessary to statistically discriminate these scenarios from the HHS instantaneous origin (i.e., in which the duration of these subpopulations would be 0), and we consider it a reasonable minimum time for a new subpopulation to genetically differentiate from its parental. The posterior distributions of these two parameters show that both reached their highest probability at the lower limit of 100 generations with a clear tendency toward even lower values (fig. S16). Setting the minimum values of *Tg* and *Ts* to 0 shows their posterior distribution tending to 0, which, in this limit, would render all scenarios equivalent to the HHS scenario (Fig. 5A). Conversely, increasing the minimum value of *Tg* and *Ts* to 300 generations increased the posterior probability of the HHS scenario to >0.99 (fig. S16).

### Estimating divergence and hybridization times

**Fig. 6.**
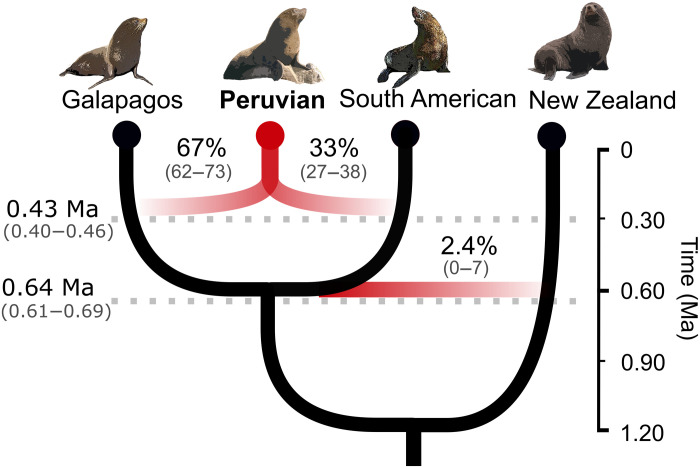
Inferred demographic parameters. Divergence times in million years (left) and ancestry percentages (above the branches) as estimated with BP&P. Below the numbers are the confidence intervals.

To estimate when the hybridization resulting in the Pfs lineage would have occurred, we used a multispecies coalescent model (MSC) in the presence of introgression implemented in BP&P program. As input, we used the topology derived from the species network with the two hybridization events obtained with the previous methods (Fig. 4, B and C) and 5000 randomly selected independent 5-kb genomic fragments. Hybridization between Gfs and SAfs giving rise to the Pfs was estimated to have occurred ~0.43 Ma (95% confidence interval: 0.40 to 0.46 Ma) with inheritance proportions of 67 and 33%, respectively (Fig. 6 and table S6). The divergence between the Gfs and SAfs was estimated at ~0.64 Ma. Approximately 2.4% of the SAfs genome was introgressed from the NZfs.

## DISCUSSION

We have used a suite of phylogenetic and population genetic methods to explore the origin of the Pfs lineage. Despite different assumptions and temporal sensitivity, all methods concurred and suggested that the Pfs is an evolutionarily independent lineage that originated from an ancient hybridization, not a recent introgression event, between the Gfs and the SAfs. Furthermore, our ABC test strongly supported the hypothesis that the Pfs originated from instantaneous HHS (Fig. 5). HHS should result in a new, differentiated, stable hybrid lineage with homogenous ancestry composition among individuals (*5*, *12*). As expected, after ancient hybridization followed by evolutionary independence for ~40,000 generations (generation length of 11.7 years) (*35*), all Pfs individuals have a distinct genetic component (Figs. 1 and 6 and fig. S13) and presented similar ancestry proportions from both putative parental species and with small ancestry blocks (Fig. 3) evenly distributed along the genome (*5*, *31*). In contrast, recent directional gene flow could also produce mixed genomes, but in this case, admixture levels are usually heterogeneous between individuals, there is no distinct genetic component, and ancestry blocks are large (*5*, *6*, *11*).

Furthermore, data from the past demographic monitoring of Pfs populations suggest that Pfs individuals are fully viable and that PSJ and other rookeries are long-term reproductive colonies. For example, in a recent census during four reproductive seasons in Peru (2016–2019), there were individuals from all age classes (pups, juveniles, subadults, and adults) in all years (*28*), showing these to be fully viable populations. In addition, since the first systematic population assessment of fur seals in Peru in 1968, Pfs rookeries have consisted of several thousand individuals (*40*). These data clearly show that Pfs individuals are viable and fertile and that Pfs is a biological entity that has existed for at least several generations, supporting our genomic results.

Some form of RI (extrinsic or intrinsic) between a new hybrid population and its putative parental species is necessary to initiate and maintain evolutionary independence (*5*, *11*). Ecogeographical isolation, instrumental in other cases of putative HHS (*15*, *41*), appears to be a central factor promoting divergence in the system: The Pfs has an allopatric distribution, ~2000 km away along the Pacific coast from each of its putative parentals, occupying an area with distinct environmental conditions (e.g., climatology; fig. S17). Thus, it is expected that the genomic results attesting to the evolutionary independence of Pfs hybrid genome align with other relevant axes of divergence (*13*). These include morphological, behavioral, and ecological differences between the Pfs and its donor species (*23*, *25*). However, the observed ancestry components in two recent hybrids (Fig. 1D and fig. S5) suggest the possibility of current but limited backcrossing between Pfs and Gfs and hence incomplete intrinsic RI between them. This is expected, since occasional hybridization is common in nature (*2*) and often evades postzygotic isolation (*42*). In the eared seals and other marine mammals, hybridization has been extensively documented (*18*, *43*, *44*), indicating poor prezygotic isolation and a role for extrinsic barriers to prevent extensive hybridization in this group.

As for what caused the ecogeographical RI of the Pfs, which involves ancient hybridization and a large organism with long generations where experimental crosses are not feasible, we must rely on indirect evidence to support the relationship between hybridization and the emergence of reproductive barriers (*12*, *15*). Each parental species is adapted to opposite environmental conditions: The Gfs is endemic to a tropical climate (living along the equator line), and the SAfs *s. str.* occurs in a temperate and much colder climate; its northernmost limit in the Pacific is in southern Chile (43°S), ~5000 km south of the Galapagos Islands (Fig. 1 and fig. S17). Besides, Gfs is adapted to large demographic oscillations during strong El Niño events (~40% population decline) (*45*, *46*), while SAfs distribution is mainly outside the area of major and direct effects of El Niño and does not experience such extreme population oscillations (*21*). In this context, the Pfs is similar to the Gfs, as it also suffers considerable population declines (>50%) during El Niño events (*46*, *47*). We suggest that the Pfs may have originated from a hybridization event between long-distant migrants from the parental species that would have met halfway, perhaps during a warmer period or El Niño event (*48*). Fur seals are highly dispersive, influenced by environmental conditions, such as marine currents and oceanographic phenomena. They can also swim long distances (>5000 km) from their reproductive sites (*49*, *50*). For example, vagrant Gfs were occasionally recorded during El Niño events along the coasts between Mexico and Ecuador, although there were no previous reports of successful breeding outside of the Galapagos Islands (*51*). However, we found that two of the three individuals sampled in Isla Foca, a small, isolated colony ~1000 km north of the Pfs main population (Fig. 1), are genetically pure Gfs juveniles, and the third seems to be a non-F1 hybrid between Gfs and Pfs. Although our sampling is still limited, these results suggest that Isla Foca is a new reproductive colony of Gfs, indicative of the geographic expansion of GFs to the continent. This demographic dynamic appears to replicate the scenario for the origin of the Pfs suggested above and may be a consequence of the recent ocean warming (*52*), which warrants further investigation. Testing these hypotheses would require whole-genome resequencing with larger sample size, including selection analyses of genes related to the ecological RI of the hybrid and its parental species (*12*).

Besides contributing to our understanding of the role of hybridization in generating species-level biodiversity, our results also have taxonomic and conservation implications. Because we show that the Pfs is an evolutionary independent taxon with unique genetic components of hybrid origin and is equally related to the Gfs and SAfs, it should not be considered a subgroup (e.g., a subspecies) of any of its parental species. Besides, the Pfs originated >0.4 Ma, a time interval of comparable magnitude to the time of speciation for other recognized pinniped species (*18*). Consequently, the correct biological and taxonomic solution would be to grant the Pfs full species status. This new status and its unique evolutionary history highlight the concern for its conservation. The Pfs is already classified as vulnerable (*47*) given its limited distribution, relatively small population size (~10,000 mature individuals), and considerable population reductions during major El Niño events (*46*). Last, genetic differentiation between the Falkland Islands and the other SAfs *s. str.* populations were low, which does not support the proposed (*53*, *54*) subspecies status for the former, as opposed to the rest of the species (Fig. 1, B and C, fig. S4, and table S3). We also did not find any substantial difference between the Pacific (Chile) and Atlantic (Brazil/Uruguay, Argentina, and Falklands) populations, in agreement with some (*32*) but contrary to other mtDNA-based results (*24*).

## MATERIALS AND METHODS

### Sample collection

In addition to the previously sequenced genomes of the NZfs, Gfs, and SAfs (from Argentina) (*18*), 69 tissue samples were collected from six populations of the SAfs along its distribution from Peru to Southern Brazil (Fig. 1 and table S2). Skin biopsies (~0.5 cm^3^) were obtained with piglet notch pliers from pups or recently dead animals found ashore, stored in 70% ethanol, and cryopreserved at −20°C. We used selective and nonlethal sampling without the application of immobilizing drugs. The appropriate catching and sampling permits were obtained from the appropriate authorities for the respective locations. Genomic DNA extractions were carried out with DNeasy Blood & Tissue Kit (QIAGEN) following the manufacturer’s protocol. DNA yield was quantified using a spectrophotometer (NanoDrop Lite), and quality was assessed on a 2% agarose gel.

### WGS sequencing

Additional new whole genome shotgun sequencing data were generated for SAfs from Peru, Chile, and Falklands (Fig. 1A and table S1). Genomic libraries were prepared with Illumina DNA polymerase chain reaction (PCR)–free or TruSeq Nano kits with an insert size of 350 base pairs (bps), and two libraries were sequenced (PE150, paired-end reads libraries of 150 bps in size generated in both directions from each end of the inserted target DNA) per lane on an Illumina HiSeq X platform. In addition, in some of our analyses, we used Illumina sequencing data associated with the genome of the Afs, *A. gazella* (*55*). Raw sequencing data for this genome were retrieved from National Center for Biotechnology Information Sequencing Read Archive (table S1).

### ddRAD-seq data generation

ddRAD-seq libraries of 72 individuals (table S2) were prepared in-house following Kess *et al*. (*56*), a modified protocol of Peterson *et al*. (*57*). Before library preparation, we performed an in silico digest based on the reference genome of the Afs (*55*), *A. gazella*, and using the python script of DaCosta and Sorenson (*58*) to estimate the expected number of RAD loci per enzyme combination. Aiming at ~15,000 genomic loci with approximately 50× coverage, the enzymes *SphI* and *Eco-RI* were selected.

For each sample, 20 ng of genomic DNA was digested with restriction enzymes. P1 and P2 barcode combinations were ligated to the generated fragments for individual identification before pooling samples. DNA fragments between 355 and 555 bps were selected using 1.5% agarose gel electrophoresis. These fragments were purified with the Invitrogen PureLink Quick Gel Extraction Kit and magnetic bead purification to remove fragments below 250 bps. Genomic libraries were sequenced on an Illumina HiSeq 2500 platform using a single-end 2× 100-bp reads module.

### Data quality control and mapping

Data quality for the different datasets was assessed with FastQC v0.11.9 software (www.bioinformatics.babraham.ac.uk/projects/fastqc, accessed 1 March 2018). The closest reference genome available to the focal clade, the Afs *A. gazella* (*55*), was included in the in-group for most of the analyses of WGS. To avoid the bias of the reference genome in genomic estimations, we used the walrus *Odobenus rosmarus* (*59*) as the reference for mapping the resequencing data of all species assessed in this study. Reads of resequencing data were mapped and locally realigned following the parameters described by Lopes *et al*. (*18*). Reads of ddRAD-seq libraries were trimmed for vestigial adapters and mapped against the closest available genome not used as an in-group in the ddRAD-seq analyses, the Afs (*55*). ddRAD-seq libraries were locally realigned using the module bam_pipeline implemented on PALEOMIX v1.2.13.2 (*60*) as follows: Reads with length size of <60 bps and sequencing quality phred score of <20 were filtered out by AdapterRemoval v2 (*61*); remaining reads were mapped using Burrows-Wheeler Aligner (BWA) v0.7.17 (*62*) and the BWA-MEM algorithm. Single-end reads with a mapping quality phred score of <20 and unmapped reads were discarded from the downstream pipeline; reads that were sequenced more than two or less than 1 SD from the average coverage of each genome were filtered out (*63*, *64*). PCR duplicates were detected and removed by Picard Tools v2.17.11 (https://broadinstitute.github.io/picard/, accessed 1 February 2018), and miscalling indels were locally realigned by Genome Analysis Toolkit v3.8 (*65*). The effective number of samples (after filters) and localities for each species are described in Fig. 1 and table S1.

SNPs were called using Analysis of Next Generation Sequencing Data (ANGSD) v0.929 (*66*). For the whole genomes, we used only sites with 100% of information; for ddRAD-seq, we removed all individuals and sites with less than 70% of genomic information (samples and sites). The distribution of missing data within the SNP panel of ddRAD-seq was graphically represented with Matrix Condenser (*67*). Detailed parameter information for filtering and mapping ddRAD-seq and whole-genome resequencing data are described in the Supplementary Text.

### Ploidy tests

To estimate the ploidy level of our focal species (Gfs, SAfs, and Pfs), we used ploidyNGS, a python program that allows the identification of ploidy level using only the sequencing reads of the WGS data (*68*). We used the parameters of minimum coverage of 10 (-u) and a maximum (-d) of 100 per site and the .bam file with the 20 largest scaffolds. The .bam file with the 20 largest scaffolds was generated with Samtools view, the options -b -h -L, and the .bed file (*69*) with the position of the 20 largest scaffolds. The results are histograms plotting the distribution of allele proportions across heterozygous positions in the genome. The number and location of peaks depend on the ploidy (and their heights on coverage and heterozygosity of the genome). For example, the expected peak frequencies are, for a diploid: 0, 0.5, and 1; for a triploid: 0, 0.33, 0.66, and 1; and for a tetraploid: 0, 0.25, 0.5, 0.75, and 1. The empirical histograms are also visually compared with simulated graphics generated with a range of ploidy levels and parameters (fig. S3B; see https://goo.gl/BHHNYM, accessed 2 March 2023).

We also evaluated the possibility of aneuploidy in the genomes using an approach that quantified the read coverage across the chromosomes (*70*). Here, we used the coverage of the 100 largest scaffolds (25% of the reference genome retrieved with Qualimap v2.2.1) (*71*). We consider this number of scaffolds sufficient to be representative of all 19 chromosomes found in the group, as their chromosomes are very similar in size: The difference between the smallest and largest is less than 5× (*30*). The regions of interest for the Qualimap analysis were generated from the mapping outputs (.bam files) with bedtools’ bamToBed function followed by the merge function (*72*). The information on the size of the scaffolds was retrieved from the index of the reference (.fai file) by sorting the size of the scaffolds in decreasing order. On the basis of this information, the 100 largest scaffolds were filtered out from the merged .bed file with bedtools intersect function and then applied to Qualimap analysis for each genome of interest. Last, the average coverage of the scaffolds was imported in a spreadsheet, where the percentage of deviation of each scaffold against the genome average was computed and the graphics generated.

### Population clustering and structure (WGS and ddRAD-seq)

First, we performed exploratory analyses to assess how individuals and genomes clustered together with a PCA using PCAngsd (see the Supplementary Text) (*73*). For the ddRAD-seq dataset, we used the ANGSD Site Frequency Spectrum to assess the weighted pairwise *F*_st_ population differentiation based on PCA clustering.

To minimize the genetic linkage and consider all SNPs as independent loci in the admixture analyzes of ddRAD-seq data, we thinned the SNP matrix to a single SNP per contig with VCFtools v0.1.16 (*69*). We evaluated the admixture proportions among individuals and populations/species using Bayesian clustering implemented in the NGSadmix ANGSD (see the Supplementary Text) (*73*). The best-fitted number of the genetic clusters was identified with cross-validation, and 10 independent replicates were calculated in admixture (from 1 to 10 clusters). The results were graphically visualized with Pophelper v2.2.9 (*74*).

### Phylogenomic inferences (WGS only)

We first used the whole-genome sequencing data to infer species trees under the MSC that account for ILS but not admixture, using StarBEAST2 (*75*) and ASTRAL-III (*76*). For these analyses, we used nonoverlapping genomic fragments of 50 kb, with a step size of 100 kb between each alignment, to avoid the effect of linkage disequilibrium (*55*). Sites with missing data and masked regions were removed with trimAl v1.4 (*77*). Scaffolds smaller than 50 kb were excluded, and only sites with 100% of the data were kept in the downstream pipeline. Alignments smaller than half of the original alignment size and noninformative alignments were discarded.

For each genomic fragment, maximum likelihood trees were computed with raxmlHPC-PTHREADS v8.2 (*78*) using GTR + G as the best-fit substitution model estimated by jModelTest2 (*79*) and Afs as the outgroup. The outputs of RAxML of each dataset were used to produce the maximum quartet support species tree (exact search) under the MSC of ASTRAL-III (*76*).

For BEAST Bayesian inference, we randomly selected 300 genomic fragments of 50 kb as described above. We obtained the root age estimation of the species tree with StarBEAST2 template implemented in the BEAST v2.5.2 package (BEAUti, BEAST, and TreeAnnotator; available from http://beast.bio.ed.ac.uk) (*75*). This analysis and the ASTRAL-III approach (see below) assume that the source of genealogical discordances is due to ILS, not considering migration events in the model. The priors used were: a strict molecular clock with a relative mutation rate = 1, linked clock models, constant population sizes, the HKY substitution model with empirical base frequencies, an estimated gamma site model, and the Yule tree model. We ran a Bayesian Markov chain Monte Carlo (MCMC) of 500,000,000 steps sampled each 20,000 with a burn-in of 10%. The divergence times were calibrated on the basis of the split time between the common ancestor of the Afs and the remaining species at 2.4 Ma. This node age was based on the calibrated whole-genome species tree of Otariidae species (*18*).

### Mitochondrial DNA sequence and phylogeny (WGS and ddRAD-seq)

Mitochondrial genomes (mtDNA) of our WGS samples were assembled by mapping raw reads with PALEOMIX v1.2.13.2 against the complete mitochondrial genome of *Arctocephalus townsendi* (GenBank NC008420) following Lopes *et al*. (*18*). These mtDNA genomes, together with those from other otariids from (*18*) or from GenBank (see fig. S6 and table S4 for more information), were automatically aligned with MUSCLE and a neighbor-joining tree estimated with MEGA X (*80*).

We also sequenced an mtDNA control region fragment of the ddRAD-seq samples of the fur seals from the Peruvian coast (PSJ and Isla Foca) as described previously (*81*) to identify the matrilineal inheritance of each sample (table S4). Identification of the mtDNA origin was based on previously published sequences (*18*, *32*, *81*, *82*).

### Topology weighting analysis (WGS only)

For Twisst analysis (topology weighting by an iterative sampling of subtrees) (*83*), we used the scripts and programs as described in https://github.com/simonhmartin/twisst (accessed 10 March 2022) and http://evomics.org/learning/population-and-speciation-genomics/2018-population-and-speciation-genomics/topology-weighting/.

### Admixture analyses (WGS only)

We used several methods to test the presence of admixture between the taxa. First, we estimated *f*_3_ and *f*_4_-statistics (*34*) with the whole genomes using threepop and fourpop modules available in the TreeMix package v1.13 (*38*, *84*). The *f*_3_-statistic explicitly tests whether a taxon of interest *C* is the result of admixture between two others (*A* and *B*), considering the product of allelic differentials between (*c* − *a*)(*c* − *b*): Negative values suggest that allelic frequencies *c* are intermediate at many populations, which is consistent with admixture. In contrast, positive results are not informative about the admixture*. f*_4_-statistics uses unrooted four-population phylogenies to visualize shared genetic drift among taxa (*84*). For an *f*_4_ (*A*,*B*;*C*,*D*) topology, without invoking admixture, the allele frequency difference between *A* and *B* (*a* − *b*) and between C and D (*c* − *d*) should be unrelated and hence results in *f*_4_ = [(*a* − *b*)(*c* − *d*)] = 0. Significantly positive *f*_4_ implies gene flow between *A* and *C* or *B* and *D*. Significantly negative *f*_4_ implies gene flow between *A* and *D* or *B* and *C*. As used here, when *A* is an outgroup, it allows testing past introgression between *B* and either *C* or *D*. A significantly positive *f*_4_ implies gene flow between *B* and *D*, and a significantly negative *f*_4_ implies gene flow between *B* and *C*. Significant *f*_4_ values may also be interpreted as rejecting the given topology (*85*, *86*). The significance of *f*_3_ and *f*_4_-statistics is based on the *z* score and was performed over 1331 blocks of 10,000 SNPs. Significantly positive (*z* > 3) and significantly negative (*z* < −3) values reject the null hypothesis (*84*). We plotted the distribution of *f*_3_ and *f*_4_ values with the function f4stats from admixturegraph (*87*), an R package.

Next, we used the Dsuite package v0.5 to estimate *D*-statistics (also called ABBA-BABA test) and the *f*_4_-ratio statistics (*35*, *88*). The *D*-statistics analysis compares the distribution of ancestral (*A*) and derived (*B*) sites in a four-taxa asymmetric phylogeny {[(P1, P2), P3], O} with P1 to P3 being in-groups and O being the outgroup. Under the null hypothesis that P1 and P2 descend from an ancestor that diverged earlier from the ancestral P3, derived alleles *B* should be found at the same proportion in P1 and P2. Gene flow between P2 and P3 will lead to an excess of ABBA patterns and a positive *D*-statistic; gene flow between P1 and P3 will lead to a surplus of BABA patterns and a negative *D*-statistics (*89*). Statistical significance for a deviation of the *D*-statistics from 0 was assessed by estimating *z* scores and their *P* values over 10,000 standard block jackknife procedures (*89*) and 1761 SNPs per block, using a *P* < 0.05 as an indication for a putative signal of genomic introgression. The *f*_4_ ratio estimates ancestry proportions in an admixed population and is calculated from the quotient of two *D*-statistics to quantify the amount of introgression in a target species [for details regarding *f*_4_ ratio, see (*34*, *90*)].

To summarize the *f*_4_ ratio in a phylogenetic context, we used the *f*-branch program in the Dsuite package. The *f*-branch (*f_b_*)*C* metric uses *f*_4_ ratio to assess gene flow evidence in branches and internal descendants of a hypothesized phylogeny. The (*f_b_*)*C* statistic reflects the excess sharing of alleles between population or taxon *C* and the descendants of the branch labeled *b* (*88*). On the basis of our phylogenetic analysis (see the Supplementary Text and Fig. 2), we used two phylogenies for the *f*-branch analyses in distinct runs: Pfs as the sister group of SAfs and Pfs as the sister group of Gfs.

### Hybrid detection with HyDe program (WGS only)

The HyDe program performs hypothesis tests on triples of taxa to detect hybridization events, including hybrid speciation, using phylogenetic invariants estimated from the genomic data (*36*). We tested all possible triples of taxa among Gfs, SAfs, and Pfs using NZfs as an outgroup. As input, we used 97,047 SNPs spaced by 25 kb. We used vcf2phylip.py with default parameters (https://github.com/edgardomortiz/vcf2phylip v2.0, accessed 11/1/2021) to transform the VCF file to the PHYLIP format as needed. We first considered all three SAfs genomes (from AR, FK, and CH) together (run_hyde.py) and next tested each SAfs individual (individual_hyde.py).

### Introgression analyses (WGS only)

Given the evidence of admixture between the taxa from the initial analyses, we next estimated the evolutionary history of the group while accounting for introgression. First, we used TreeMix, a method that estimates the patterns of population splits and historical events of admixture by using a set of SNPs of extant populations. Next, we used admixtools2 in R (https://uqrmaie1.github.io/admixtools, accessed 11 January 2021) to estimate admixture graphs. The SNP dataset used above for the Dsuite analyses was thinned to one SNP each 25 kb, resulting in 96,026 SNPs with no missing data. The best-fit graph was estimated using five independent runs of command find_graphs(stop_gen2 = 1000, numgraphs = 100, plusminus_generations = 20). Confidence intervals were estimated using qpgraph_resample_snps with 100 bootstraps. The above methods used statistics (such as *f*_2_, *f*_3_, and *D*-statistics) estimated from individual SNPs.

We also inferred phylogenetic networks (that account for ILS and introgression) with PhyloNet v3.8.2 (*91*) command MCMC_GT, which uses gene trees to perform Bayesian inference of the posterior distribution of the species network. We used 7810 gene trees from genomic fragments of 50 kb separated by 100 kb (see the Supplementary Text). To reduce the effect of intrafragment genetic recombination on the phylogenetic estimates, we used the software 3SEQ on full run mode (*92*) and removed the alignments with evidence of recombination at a *P <* 0.01 after Bonferroni correction (*93*). We ran an MCMC chain with a length of 5 × 10^7^ iterations sampled every 5000 steps, discarding the first 1000 as burn-in with the pseudo-likelihood option (instead of full likelihood) and a maximum of three reticulations to reduce runtime. On the basis of the previous analysis (i.e., TreeMix, *f*-branch, PCA, and *F*_ST_), we considered SAfs from Argentina, Falklands, and Chile as a unique taxon. The hybridization network tree was visualized with Dendroscope v3.7.4.

### Divergence times (WGS only)

To estimate divergence and introgression times, we used a Bayesian MCMC method with a MSC model in the presence of introgression (MSCi) as implemented in BP&P v4.1.4 (*94*). We used 5000 randomly selected genomic fragments of 5 kb each generated with the pipeline described in the Supplementary Text. The genomic fragments used were filtered to maximize the amount of fully recombined, independent segments in the same way as mentioned above for the PhyloNet MCMC_GT analysis. In the BP&P run, we used module A00 to estimate the divergence times under the MSCi model when the species network is given (*94*, *95*) to allow acceptable runtimes. We used the network with the maximum posterior value estimated by PhyloNet MCMC_GT, allowing migration among Pfs-Gfs-SAfs and between SAfs-NZfs. The MCMC was run for 8 × 10^5^ iterations after 2 × 10^4^ iterations of burn-in with theta and tau prior set to 2 × 10^−3^. The theta prior specifies the inverse gamma prior, the number of differences per kilobyte, and the tau specifies the divergence time parameter for the root (*18*). Samples were recorded every four iterations. The convergence of the MCMC runs was assessed with Tracer v1.7.1. For this analysis, the divergence times were also calibrated on the basis of the age of the root from Lopes *et al*. (*18*).

### Test of hybridization scenarios with DIYABC (ddRAD-seq only)

We used the approximate Bayesian computation method implemented on DIYABC v2.1.0 (*96*) to test the most likely evolutionary hybrid scenario for the origin of Pfs with the ddRAD-seq SNPs dataset. We tested four alternative scenarios for hybridization, depicted in Fig. 5 and fig. S14. Scenario 1 is an HHS, in which individuals from the Gfs and SAfs hybridize to produce the Pfs without forming previously differentiated lineages. Alternative, non-HHS scenarios are introgression from Gfs into a previously isolated population of SAfs originating the Pfs or from SAfs into a previously isolated population of Gfs originating the Pfs (scenarios 2 and 3, respectively) and a hybrid origin of Pfs from the fusion of two previously isolated populations from Gfs and SAfs (scenario 4). Note that scenario 4 may also be considered an HHS model. We did not include the NZfs genome in the analyses to maintain simpler scenarios and improve estimation efficiency.

The distribution of the priors for the parameters is presented in fig. S14. The population size priors for the three species were set to a large interval but those for the ancestral populations that ultimately originated the Pfs (in scenarios 2 to 4) were narrower (10 to 10,000). As priors, for the time of the divergence of Gfs and Pfs and the hybridization, we used the estimates from the BP&P analyses. For the non-HHS (2, 3, and 4) scenarios, we first set a minimum of 100 generations between the origin of the population(s) and the introgression or hybridization that originated the Pfs (parameters *Tg* and *Ts*). We consider this a reasonable minimum time for an offshoot population to be considered a genetically different population from its parent and for the method to be able to statistically differentiate these scenarios from the HHS “instantaneous” origin (i.e., without the formation of a previously differentiated population). However, to test the effect of this limitation on our results, we ran two additional analyses: one analysis with *Tg* and *Ts* set to a minimum of 300 generations and the other setting these parameters to a minimum of 0. Note that as *Tg* and *Ts* approached 0, this essentially renders the alternative scenarios 2 to 4 equivalent to the HHS scenario.

All available summary statistics were used. We performed 1,000,000 simulations for each scenario in every run above. The posterior probability of each scenario (scenario choice) was computed with the logistic regression based on the 40,000 simulated data closest to the observed data and the four intermediate values. In addition, pre-evaluation scenario-prior combinations and model checking for the best scenario were performed with PCA with the default options. Model checking, as described in DIYABC manual, “is a PCA in the space of summary statistics using datasets simulated with the prior distributions of parameters and the observed data as well as datasets from the posterior predictive distribution that are represented on each plane of the PCA. If the model fits the data well, then one should see on each PCA plane a wide cloud of datasets simulated from the prior, with the observed dataset in the middle of a small cluster of datasets from the posterior predictive distribution.” The settings for this analysis were logit transformation of parameters on 10,000 simulated data closest to the observed data and 1000 datasets simulated from the posterior. We used DIYABC analyses only to test the evolutionary scenarios, not to estimate the time parameters; as in DIYABC with ddRAD-seq SNP dataset, absolute times are not informative (see the DIYABC v2.1.0 manual). We additionally estimated the posterior distribution of the *Tg* and *Ts* parameters, as they are the ones that allow us to better understand the differentiation between the HHS and the introgression scenarios.

## Supplementary Materials

### This PDF file includes:

Supplementary Text Figs. S1 to S17 Tables S1 to S6 References

## Notes

View/request a protocol for this paper from *Bio-protocol*.

## References

[1] J. Mallet, Hybrid speciation. Nature 446, 279–283 (2007). 17361174 10.1038/nature05706

[2] J. Mallet, Hybridization as an invasion of the genome. Trends Ecol. Evol. 20, 229–237 (2005). 16701374 10.1016/j.tree.2005.02.010

[3] R. Abbott, D. Albach, S. Ansell, J. W. Arntzen, S. J. E. Baird, N. Bierne, J. Boughman, A. Brelsford, C. A. Buerkle, R. Buggs, R. K. Butlin, U. Dieckmann, F. Eroukhmanoff, A. Grill, S. H. Cahan, J. S. Hermansen, G. Hewitt, A. G. Hudson, C. Jiggins, J. Jones, B. Keller, T. Marczewski, J. Mallet, P. Martinez-Rodriguez, M. Möst, S. Mullen, R. Nichols, A. W. Nolte, C. Parisod, K. Pfennig, A. M. Rice, M. G. Ritchie, B. Seifert, C. M. Smadja, R. Stelkens, J. M. Szymura, R. Väinölä, J. B. W. Wolf, D. Zinner, Hybridization and speciation. J. Evol. Biol. 26, 229–246 (2013). 23323997 10.1111/j.1420-9101.2012.02599.x

[4] A. Runemark, C. N. Trier, F. Eroukhmanoff, J. S. Hermansen, M. Matschiner, T. O. Elgvin, G. P. Sætre, Variation and constraints in hybrid genome formation. Nat. Ecol. Evol. 2, 549–556 (2018). 29335572 10.1038/s41559-017-0437-7

[5] A. Runemark, M. Vallejo-Martin, J. I. Meier, Eukaryote hybrid genomes. PLOS Genet. 15, e1008404 (2019). 31774811 10.1371/journal.pgen.1008404 PMC6880984

[6] R. J. Abbott, N. H. Barton, J. M. Good, Genomics of hybridization and its evolutionary consequences. Mol. Ecol. 25, 2325–2332 (2016). 27145128 10.1111/mec.13685

[7] D. A. Marques, J. Meier, O. Seehausen, A combinatorial view on speciation and adaptive radiation. Trends Ecol. Evol. 34, 531–544 (2019). 30885412 10.1016/j.tree.2019.02.008

[8] A. W. Nolte, D. Tautz, Understanding the onset of hybrid speciation. Trends Genet. 26, 54–58 (2010). 20044166 10.1016/j.tig.2009.12.001

[9] N. B. Edelman, J. Mallet, Prevalence and adaptive impact of introgression. Annu. Rev. Genet. 55, 265–283 (2021). 34579539 10.1146/annurev-genet-021821-020805

[10] G. Nieto Feliner, I. Álvarez, J. Fuertes-Aguilar, M. Heuertz, I. Marques, F. Moharrek, R. Piñeiro, R. Riina, J. A. Rosselló, P. S. Soltis, I. Villa-Machío, Is homoploid hybrid speciation that rare? An empiricist’s view. Heredity 118, 513–516 (2017). 28295029 10.1038/hdy.2017.7 PMC5436029

[11] M. Schumer, G. G. Rosenthal, P. Andolfatto, How common is homoploid hybrid speciation? Evolution 68, 1553–1560 (2014). 24620775 10.1111/evo.12399

[12] Z. Wang, M. Kang, J. Li, Z. Zhang, Y. Wang, C. Chen, Y. Yang, J. Liu, Genomic evidence for homoploid hybrid speciation between ancestors of two different genera. Nat. Commun. 13, 1987 (2022). 35418567 10.1038/s41467-022-29643-4 PMC9008057

[13] D. Bolnick, A. K. Hund, P. Nosil, F. Peng, M. Ravinet, S. Stankowski, S. Subramanian, J. B. W. Wolf, R. Yukilevich, A multivariate view of the speciation continuum. Evolution 1, 318–328 (2023). 10.1093/evolut/qpac004 36622661

[14] Y. Sun, Z. Lu, X. Zhu, H. Ma, Genomic basis of homoploid hybrid speciation within chestnut trees. Nat. Commun. 11, 3375 (2020). 32632155 10.1038/s41467-020-17111-w PMC7338469

[15] D. Ru, Y. Sun, D. Wang, Y. Chen, T. Wang, Q. Hu, R. J. Abbott, J. Liu, Population genomic analysis reveals that homoploid hybrid speciation can be a lengthy process. Mol. Ecol. 27, 4875–4887 (2018). 30357974 10.1111/mec.14909

[16] S. A. Taylor, E. L. Larson, Insights from genomes into the evolutionary importance and prevalence of hybridization in nature. Nat. Ecol. Evol. 3, 170–177 (2019). 30697003 10.1038/s41559-018-0777-y

[17] R. Adavoudi, M. Pilot, Consequences of hybridization in mammals: A systematic review. Genes 13, 50 (2022). 10.3390/genes13010050 PMC8774782 35052393

[18] F. Lopes, L. R. Oliveira, A. Kessler, Y. Beux, E. Crespo, S. Cárdenas-Alayza, P. Majluf, M. Sepúlveda, R. L. Brownell Jr., V. Franco-Trecu, D. Páez-Rosas, J. Chaves, C. Loch, B. C. Robertson, K. Acevedo-Whitehouse, F. R. Elorriaga-Verplancken, S. P. Kirkman, C. R. Peart, J. B. W. Wolf, S. L. Bonatto, Phylogenomic discordance in the eared seals is best explained by incomplete lineage sorting following explosive radiation in the Southern Hemisphere. Syst. Biol. 70, 786–802 (2021). 33367817 10.1093/sysbio/syaa099

[19] F. Trillmich, *Arctocephalus galapagoensis*. *The IUCN Red List of Threatened Species*. 2015:e.T2057A45223722 (2015); 10.2305/IUCN.UK.2015-2.RLTS.T2057A45223722.en.

[20] B. L. Chilvers, S. D. Goldsworthy, *Arctocephalus forsteri*. *The IUCN Red List of Threatened Species*. 2015. e.T41664A45230026 (2015); 10.2305/IUCN.UK.2015-2.RLTS.T41664A45230026.en.

[21] S. Cárdenas-Alayza, L. Oliveira, E. Crespo, *Arctocephalus australis*. *The IUCN Red List of Threatened Species*. e.T2055A45223529 (2016).

[22] L. Oliveira, E. Hingst-Zaher, J. S. Morgante, Size and shape sexual dimorphism in the skull of the South American fur seal, *Arctocephalus australis* (Zimmermann, 1783) (Carnivora: Otariidae). Lat. Am. J. Aquat. Mamm. 4, 27–40 (2005).

[23] L. R. de Oliveira, J. I. Hoffman, E. Hingst-Zaher, P. Majluf, M. M. C. Muelbert, J. S. Morgante, W. Amos, Morphological and genetic evidence for two evolutionarily significant units (ESUs) in the South American fur seal, *Arctocephalus gazella*. Conserv. Genet. 9, 1451–1466 (2008).

[24] J. I. Túnez, H. L. Cappozzo, H. Paves, D. A. Albareda, M. H. Cassini, The role of Pleistocene glaciations in shaping the genetic structure of South American fur seals (*Arctocephalus australis*). N. Z. J. Mar. Freshw. Res. 47, 139–152 (2013).

[25] H. J. Pavés, R. P. Schlatter, V. Franco-Trecu, E. Paez, W. Siefeld, V. Araos, R. Giesecke, L. M. Batallés, H. L. Capozzo, Breeding season of the South American fur seal (*Arctocephalus australis*, Otariidae: Carnivora): New data for establishing independent evolutionary histories? Rev. Biol. Mar. Oceanogr. 51, 241–253 (2016).

[26] A. Berta, M. Churchill, Pinniped taxonomy: Review of currently recognized species and subspecies, and evidence used for their description. Mamm. Rev. 42, 207–234 (2012).

[27] L. R. de Oliveira, R. L. Brownell Jr., Taxonomic status of two subspecies of South American fur seals: *Arctocephalus australis australis* vs. *A. a. gracilis*. Mar. Mamm. Sci. 30, 1258–1263 (2014).

[28] R. Aguilar-Arakaki, Población del lobo fino *Arctocephalus australis* en la costa peruana en el periodo 2016-2019. Bol. Inst. Mar. Perú 36, 188–204 (2021).

[29] B. J. Evans, N. S. Upham, G. B. Golding, R. A. Ojeda, A. A. Ojeda, Evolution of the largest mammalian genome. Genome Biol. Evol. 9, 1711–1724 (2017). 28854639 10.1093/gbe/evx113 PMC5569995

[30] A. Beilts, I. M. Rahn, M. C. G. Moreno, J. Loureiro, M. S. Merani, Mitotic and meiotic analysis in *Arctocephalus australis* (Otariidae). Hereditas 131, 33–37 (1999). 10628295 10.1111/j.1601-5223.1999.t01-1-00033.x

[31] T. O. Elgvin, C. N. Trier, O. K. Tørresen, I. J. Hagen, S. Lien, A. J. Nederbragt, M. Ravinet, H. Jensen, G.-P. Sætre, The genomic mosaicism of hybrid speciation. Sci. Adv. 3, e1602996 (2017). 28630911 10.1126/sciadv.1602996 PMC5470830

[32] P. Rodrigues, M. Seguel, J. Gutiérrez, H. Pavés, C. Verdugo, Genetic connectivity of the South American fur seal (*Arctocephalus australis*) across Atlantic and Pacific oceans revealed by mitochondrial genes. Aquat. Conserv. Mar. Freshw. Ecosyst. 28, 315–323 (2018).

[33] T. Yonezawa, N. Kohno, M. Hasegawa, The monophyletic origin of sea lions and fur seals (Carnivora; Otariidae) in the Southern Hemisphere. Gene 441, 89–99 (2009). 19254754 10.1016/j.gene.2009.01.022

[34] N. Patterson, P. Moorjani, Y. Luo, S. Mallick, N. Rohland, Y. Zhan, T. Genschoreck, T. Webster, D. Reich, Ancient admixture in human history. Genetics 192, 1065–1093 (2012). 22960212 10.1534/genetics.112.145037 PMC3522152

[35] M. Malinsky, M. Matschine, H. Svardal, Dsuite - Fast D-statistics and related admixture evidence from VCF files. Mol. Ecol. Res. 21, 584–595 (2021). 10.1111/1755-0998.13265 PMC7116594 33012121

[36] P. D. Blischak, J. Chifman, A. D. Wolfe, L. S. Kubatko, HyDe: A Python package for genome-scale hybridization detection. Syst. Biol. 67, 821–829 (2018). 29562307 10.1093/sysbio/syy023 PMC6454532

[37] S. Kong, L. S. Kubatko, Comparative performance of popular methods for hybrid detection using genomic data. Syst. Biol. 70, 891–907 (2021). 33404632 10.1093/sysbio/syaa092

[38] J. K. Pickrell, J. K. Pritchard, Inference of population splits and mixtures from genome-wide allele frequency data. PLOS Genet. 8, e1002967 (2012). 23166502 10.1371/journal.pgen.1002967 PMC3499260

[39] S. M. Boca, L. Huang, N. A. Rosenberg, On the heterozygosity of an admixed population. J. Math. Biol. 81, 1217–1250 (2020). 33034736 10.1007/s00285-020-01531-9 PMC7710588

[40] P. Majluf, F. Trillmich, Distribution and abundance of sea lions (*Otaria byronia*) and fur seals (*Arctocephalus australis*) in Peru. Zeitschrift für Säugetierkunde 46, 384–393 (1981).

[41] A. G. Fabritzek, E. M. Griebeler, J. W. Kadereit, Hybridization, ecogeographical displacement and the emergence of new lineages–A genotyping-by-sequencing and ecological niche and species distribution modelling study of *Sempervivum tectorum* L. (Houseleek). J. Evol. Biol. 34, 830–844 (2021). 33714223 10.1111/jeb.13784

[42] T. D. Price, M. M. Bouvier, The evolution of F_1_ postzygotic incompatibilities in birds. Evolution 56, 2083–2089 (2002). 12449494

[43] M. L. Lancaster, N. J. Gemmell, S. Negro, S. Goldsworthy, P. Sunnucks, Ménage à trois on Macquarie Island: Hybridization among three species of fur seal (*Arctocephalus* spp.) following historical population extinction. Mol. Ecol. 15, 3681–3692 (2006). 17032266 10.1111/j.1365-294X.2006.03041.x

[44] M. N. Schaurich, F. Lopes, L. R. Oliveira, Hybridization phenomenon in cetacean and pinniped species. Neotrop. Biol. Conserv. 7, 199–209 (2012).

[45] D. Páez-Rosas, J. Torres, E. Espinoza, A. Marchetti, H. Seim, M. Riofrío-Lazo, Declines and recovery in endangered Galapagos pinnipeds during the El Niño event. Sci. Rep. 11, 8785 (2021). 33888850 10.1038/s41598-021-88350-0 PMC8075323

[46] L. R. de Oliveira, D. Meyer, J. Hoffman, P. Majluf, J. S. Morgante, Evidence of a genetic bottleneck in an El Niño affected population of South American fur seals, *Arctocephalus australis*. J. Mar. Biol. Assoc. U.K. 89, 1717–1725 (2009).

[47] S. Cárdenas-Alayza, L. Oliveira, *Arctocephalus australis* (Peruvian/Northern Chilean subpopulation. *The IUCN Red List of Threatened Species*. e.T72050476A72050985 (2016).

[48] G. T. Rustic, P. J. Polissar, A. C. Ravelo, S. M. White, Modulation of late Pleistocene ENSO strength by the tropical Pacific thermocline. Nat. Commun. 11, 5377 (2020). 33097727 10.1038/s41467-020-19161-6 PMC7584583

[49] L. Milmann, R. Machado, L. R. de Oliveira, P. H. Ott, Far away from home: Presence of fur seal (*Arctocephalus* sp.) in the equatorial Atlantic Ocean. Polar Biol. 42, 817–822 (2019).

[50] D. Páez-Rosas, D. Pazmiño, M. Riofrío-Lazo, Unprecedented records of Guadalupe and Juan Fernández fur seals in the Galapagos archipelago. Aquat. Mamm. 46, 549–555 (2020).

[51] D. Páez-Rosas, L. A. Valdovinos, F. R. Elorriaga-Verplancken, Northernmost record of the Galapagos fur seal (*Arctocephalus galapagoensis*): A consequence of anomalous warm conditions around the Galapagos Archipelago. Aquat. Mamm. 43, 629–634 (2017).

[52] E. S. Poloczanska, M. T. Burrows, C. J. Brown, J. García Molinos, B. S. Halpern, O. Hoegh-Guldberg, C. V. Kappel, P. J. Moore, A. J. Richardson, D. S. Schoeman, W. J. Sydeman, Responses of marine organisms to climate change across oceans. Front. Mar. Sci. 3, 62 (2016).

[53] J. E. King, The otariid seals of the Pacific coast of America in *Bull British Mus* (Natural History, 1954), vol. 2, pp. 309–337.

[54] J. E. King, *Seals of the world* (Cornell University Press, Ithaca, NY, ed. 2nd, 1983).

[55] E. Humble, K. K. Dasmahapatra, A. Martinez-Barrio, I. Gregório, J. Forcada, A.-C. Polikeit, S. D. Goldsworthy, M. E. Goebel, J. Kalinowski, J. B. W. Wolf, J. I. Hoffman, RAD sequencing and a hybrid Antarctic fur seal genome assembly reveal rapidly decaying linkage disequilibrium, global population structure and evidence for inbreeding. G3 (Bethesda) 8, 2709–2722 (2018). 29954843 10.1534/g3.118.200171 PMC6071602

[56] T. Kess, J. Gross, F. Harper, E. G. Boulding, Low-cost ddRAD method of SNP discovery and genotyping applied to the periwinkle *Littorina saxatilis*. J. Mollus. Stud. 82, 104–109 (2016).

[57] B. K. Peterson, J. N. Weber, E. H. Kay, H. S. Fisher, H. E. Hoekstra, Double digest RADseq: An inexpensive method for de novo SNP discovery and genotyping in model and non-model species. PLOS ONE 7, e37135 (2012). 22675423 10.1371/journal.pone.0037135 PMC3365034

[58] J. M. DaCosta, M. D. Sorenson, Amplification biases and consistent recovery of loci in a double-digest RAD-seq protocol. PLOS ONE 9, e106713 (2014). 25188270 10.1371/journal.pone.0106713 PMC4154734

[59] A. D. Foote, Y. Liu, G. W. C. Thomas, T. Vinař, J. Alföldi, J. Deng, S. Dugan, C. E. van Elk, M. E. Hunter, V. Joshi, Z. Khan, C. Kovar, S. L. Lee, K. Lindblad-Toh, A. Mancia, R. Nielsen, X. Qin, J. Qu, B. J. Raney, N. Vijay, J. B. W. Wolf, M. W. Hahn, D. M. Muzny, K. C. Worley, M. T. P. Gilbert, R. A. Gibbs, Convergent evolution of the genomes of marine mammals. Nat. Genet. 47, 272–275 (2015). 25621460 10.1038/ng.3198 PMC4644735

[60] M. Schubert, L. Ermini, C. D. Sarkissian, H. Jónsson, A. Ginolhac, R. Schaefer, M. D. Martin, R. Fernández, M. Kircher, M. McCue, E. Willerslev, L. Orlando, Characterization of ancient and modern genomes by SNP detection and phylogenomic and metagenomic analysis using PALEOMIX. Nat. Protoc. 9, 1056–1082 (2014). 24722405 10.1038/nprot.2014.063

[61] M. Schubert, S. Lindgreen, L. Orlando, AdapterRemoval v2: Rapid adapter trimming, identification, and read merging. BMC Res. Notes 9, 88 (2016). 26868221 10.1186/s13104-016-1900-2 PMC4751634

[62] H. Li, R. Durbin, Fast and accurate short read alignment with Burrows-Wheeler transform. Bioinformatics 25, 1754–1760 (2009). 19451168 10.1093/bioinformatics/btp324 PMC2705234

[63] B. Arnold, R. B. Corbett-Detig, D. Hartl, K. Bomblies, RADseq underestimates diversity and introduces genealogical biases due to nonrandom haplotype sampling. Mol. Ecol. 22, 3179–3190 (2013). 23551379 10.1111/mec.12276

[64] M. Gautier, K. Gharbi, T. Cezard, J. Foucaud, C. Kerdelhué, P. Pudlo, J.-M. Cornuet, A. Estoup, The effect of RAD allele dropout on the estimation of genetic variation within and between populations. Mol. Ecol. 22, 3165–3178 (2013). 23110526 10.1111/mec.12089

[65] A. McKenna, M. Hanna, E. Banks, A. Sivachenko, K. Cibulskis, A. Kernytsky, K. Garimella, D. Altshuler, S. Gabriel, M. Daly, M. A. DePristo, The Genome Analysis Toolkit: A MapReference framework for analyzing next-generation DNA sequencing data. Genome Res. 20, 1297–1303 (2010). 20644199 10.1101/gr.107524.110 PMC2928508

[66] T. S. Korneliussen, A. Albrechtsen, R. Nielsen, ANGSD: Analysis of Next Generation Sequencing Data. BMC Bioinformatics 15, 356 (2014). 25420514 10.1186/s12859-014-0356-4 PMC4248462

[67] B. A. S. de Medeiros, B. D. Farrell, Whole-genome amplification in double-digest RADseq results in adequate libraries but fewer sequenced loci. PeerJ. 6, e5089 (2018). 30038852 10.7717/peerj.5089 PMC6054070

[68] R. A. C. D. Santos, G. H. Goldman, D. M. Riaño-Pachón, ploidyNGS: Visually exploring ploidy with Next Generation Sequencing data. Bioinformatics 33, 2575–2576 (2017). 28383704 10.1093/bioinformatics/btx204

[69] P. Danecek, A. Auton, G. Abecasis, C. A. Albers, E. Banks, M. A. DePristo, R. E. Handsaker, G. Lunter, G. T. Marth, S. T. Sherry, G. McVean, R. Durbin; 1000 Genomes Project Analysis Group, The variant call format and VCFtools. Bioinformatics 27, 2156–2158 (2011). 21653522 10.1093/bioinformatics/btr330 PMC3137218

[70] N. Singh, J. Raupp, D.-H. Koo, B. Friebe, B. Gill, J. Poland, In-silico detection of aneuploidy and chromosomal deletions in wheat using genotyping-by-sequencing. Plant Methods 16, 45 (2020). 32280361 10.1186/s13007-020-00588-3 PMC7137276

[71] K. Okonechnikov, A. Conesa, F. García-Alcalde, Qualimap 2: Advanced multi-sample quality control for high-throughput sequencing data. Bioinformatics 32, 292–294 (2016). 26428292 10.1093/bioinformatics/btv566 PMC4708105

[72] A. R. Quinlan, I. M. Hall, BEDTools: A flexible suite of utilities for comparing genomic features. Bioinformatics 26, 841–842 (2010). 20110278 10.1093/bioinformatics/btq033 PMC2832824

[73] J. Meisner, A. Albretchtsen, Inferring population structure and admixture proportions in low-depth NGS data. Genetics 210, 719–731 (2018). 30131346 10.1534/genetics.118.301336 PMC6216594

[74] R. M. Francis, Pophelper: An R package and web app to analyse and visualize population structure. Mol. Ecol. Resour. 17, 27–32 (2017). 26850166 10.1111/1755-0998.12509

[75] H. A. Ogilvie, R. R. Bouckaert, A. J. Drummond, StarBEAST2 Brings faster species tree inference and accurate estimates of substitution rates. Mol. Biol. Evol. 34, 2101–2114 (2017). 28431121 10.1093/molbev/msx126 PMC5850801

[76] C. Zhang, M. Rabiee, E. Sayyari, S. Mirarab, ASTRAL-III: Polynomial time species tree reconstruction from partially resolved gene trees. BMC Bioinformatics 19, 153 (2018). 29745866 10.1186/s12859-018-2129-y PMC5998893

[77] S. Capella-Gutiérrez, J. M. Silla-Martínez, T. Gabaldón, trimAl: A tool for automated alignment trimming in large-scale phylogenetic analyses. Bioinformatics 25, 1972–1973 (2009). 19505945 10.1093/bioinformatics/btp348 PMC2712344

[78] A. Stamatakis, RAxML version 8: A tool for phylogenetic analysis and post-analysis of large phylogenies. Bioinformatics 30, 1312–1313 (2014). 24451623 10.1093/bioinformatics/btu033 PMC3998144

[79] D. Darriba, G. L. Taboada, R. Doallo, D. Posada, jModelTest 2: More models, new heuristics and parallel computing. Nat. Methods 9, 772 (2012). 10.1038/nmeth.2109 PMC4594756 22847109

[80] S. Kumar, G. Stecher, M. Li, C. Knyaz, K. Kumar, Mega X: Molecular Evolutionary Genetics Analysis across computing platforms. Mol. Biol. Evol. 35, 1547–1549 (2018). 29722887 10.1093/molbev/msy096 PMC5967553

[81] F. Lopes, J. I. Hoffman, V. H. Valiati, S. L. Bonatto, J. B. W. Wolf, F. Trillmich, L. R. Oliveira, Fine-scale matrilineal population structure in the Galapagos fur seal and its implications for conservation management. Conserv. Genet. 16, 1099–1113 (2015).

[82] L. P. Wynen, S. D. Goldsworthy, S. J. Insley, M. Adams, J. W. Bickham, J. Francis, J. P. Gallo, A. R. Hoelzel, P. Majluf, R. W. G. White, R. Slade, Phylogenetic relationships within the eared seals (Otariidae: Carnivora): Implications for the historical biogeography of the family. Mol. Phylogenet. Evol. 21, 270–284 (2001). 11697921 10.1006/mpev.2001.1012

[83] S. H. Martin, S. M. Van Belleghem, Exploring evolutionary relationships across the genome using topology weighting. Genetics 206, 429–438 (2017). 28341652 10.1534/genetics.116.194720 PMC5419486

[84] A. M. Harris, M. De Giorgio, Admixture and ancestry inference from ancient and modern samples through measures of population genetic drift. Hum. Biol. 89, 21–46 (2017). 29285965 10.13110/humanbiology.89.1.02

[85] B. M. Peter, Admixture, population structure, and F-statistics. Genetics 202, 1485–1501 (2016). 26857625 10.1534/genetics.115.183913 PMC4905545

[86] Y. Zheng, A. Janke, Gene flow analysis method, the D-statistic, is robust in a wide parameter space. BMC Bioinformatics 19, 10 (2018). 29310567 10.1186/s12859-017-2002-4 PMC5759368

[87] K. Leppälä, S. V. Nielsen, T. Mailund, admixturegraph: An R package for admixture graph manipulation and fitting. Bioinformatics 33, 1738–1740 (2017). 28158333 10.1093/bioinformatics/btx048 PMC5447235

[88] M. Malinsky, H. Svardal, A. M. Tyers, E. A. Miska, M. J. Genner, G. F. Turner, R. Durbin, Whole-genome sequences of Malawi cichlids reveal multiple radiations interconnected by gene flow. Nat. Ecol. Evol. 2, 1940–1955 (2018). 30455444 10.1038/s41559-018-0717-x PMC6443041

[89] E. Y. Durand, N. Patterson, D. Reich, M. Slatkin, Testing for ancient admixture between closely related populations. Mol. Biol. Evol. 28, 2239–2252 (2011). 21325092 10.1093/molbev/msr048 PMC3144383

[90] D. Reich, R. E. Green, M. Kircher, J. Krause, N. Patterson, E. Y. Durand, B. Viola, A. W. Briggs, U. Stenzel, P. L. Johnson, T. Maricic, J. M. Good, T. Marques-Bonet, C. Alkan, Q. Fu, S. Mallick, H. Li, M. Meyer, E. E. Eichler, M. Stoneking, M. Richards, S. Talamo, M. V. Shunkov, A. P. Derevianko, J.-J. Hublin, J. Kelso, M. Slatkin, S. Pääbo, Genetic history of an archaic hominin group from Denisova Cave in Siberia. Nature 468, 1053–1060 (2010). 21179161 10.1038/nature09710 PMC4306417

[91] D. Wen, Y. Yu, J. Zhu, L. Nakhleh, Inferring phylogenetic networks using PhyloNet. Syst. Biol. 67, 735–740 (2018). 29514307 10.1093/sysbio/syy015 PMC6005058

[92] H. M. Lam, O. Ratmann, M. F. Boni, Improved algorithmic complexity for the 3SEQ recombination detection algorithm. Mol. Biol. Evol. 35, 247–251 (2018). 29029186 10.1093/molbev/msx263 PMC5850291

[93] W. R. Rice, Analyzing tables of statistical tests. Evolution 43, 223–225 (1989). 28568501 10.1111/j.1558-5646.1989.tb04220.x

[94] T. Flouri, X. Jiao, B. Rannala, Z. Yang, A Bayesian implementation of the multispecies coalescent model with introgression for phylogenomic analysis. Mol. Biol. Evol. 37, 1211–1223 (2020). 31825513 10.1093/molbev/msz296 PMC7086182

[95] B. Rannala, Z. Yang, Bayes estimation of species divergence times and ancestral population sizes using DNA sequences from multiple loci. Genetics 164, 1645–1656 (2003). 12930768 10.1093/genetics/164.4.1645 PMC1462670

[96] J.-M. Cornuet, P. Pudlo, J. Veyssier, A. Dehne-Garcia, M. Gautier, R. Leblois, J.-M. Marin, A. Estoup, DIYABC v2.0: A software to make approximate Bayesian computation inferences about population history using single nucleotide polymorphism, DNA sequence and microsatellite data. Bioinformatics 30, 1187–1189 (2014). 24389659 10.1093/bioinformatics/btt763

[97] C. C. Chang, C. C. Chow, L. C. Tellier, S. Vattikuti, S. M. Purcell, J. J. Lee, Second-generation PLINK: Rising to the challenge of larger and richer datasets. GigaScience 4, 7 (2015). 25722852 10.1186/s13742-015-0047-8 PMC4342193

