# Supplementary material for: Genomic evidence for homoploid hybrid speciation in a marine mammal apex predator
Source: Sci Adv. 2023 May 3;9(18):eadf6601. doi: 10.1126/sciadv.adf6601 (PMC10156116; doi:10.1126/sciadv.adf6601)
Supplement: Supplementary file 1 — Supplementary Text Figs. S1 to S17 Tables S1 to S6 References [file sciadv.adf6601_sm.pdf]

Science Advances
AAAS

Supplementary Materials for

Genomic evidence for homoploid hybrid speciation in a marine mammal apex predator

Fernando Lopes *et al.*

Corresponding author: Sandro L. Bonatto, slbonatto@pucrs.br; Fernando Lopes, fernando.lopes@edu.pucrs.br

Sci. Adv. **9**, eadf6601 (2023)
DOI: 10.1126/sciadv.adf6601

This PDF file includes:

Supplementary Text
Figs. S1 to S17
Tables S1 to S6
References

Supplementary Text

SNP calling and filtering parameters

ddRAD-seq parameters:

SNPs were called with ANGSD 0.921 (*66*) using the parameters:

doPlink 2, uniqueOnly 1, remove_bads 1, only_proper_pairs 0, C 50, baq 1, setMinDepth 235, setMaxDepth 4700, setMinDepthInd 5, setMaxDepthInd 100, doCounts 1, GL 2, doMajorMinor 1, doGlf 2, SNP_pval 1e-3, minInd 38, doGeno 4, doPost 1, doMaf, postCutoff 0.99, geno_minDepth 4. After the SNP calling, the variant panel was converted to VCF format with Plink 1.9 (*97*).

Resequencing data parameters:

From the mapped reads, SNPs were called also using ANGSD 0.921 (*66*) with the parameters: *doFasta 2*, *doCounts1* and *explode 1*. Single-nucleotide polymorphisms (SNPs) were called with settings: *uniqueOnly 1*, *remove_bads 1*, *only_proper_pairs 1*, *C 50, baq 1*, *setMinDepth* 120, *setMaxDepth 1200*, *setMinDepthInd 5*, *setMaxDepthInd 100*, *doCounts 1, GL 2*, *doMajorMinor 1*, *SNP_pval 1e-3*, *doGeno 32*, *doPost 1, doPlink2*. After the SNP calling, the variant panel was converted to VCF format with Plink 1.9 (*97*)

Parameters meaning:

Quality filtering

- only_proper_pairs =1: include only proper pairs (pairs of read with both mates mapped correctly). 1: include only proper (default), 0: use all reads. Only relevant for paired end data;
- -C 50: adjust mapQ for excessive mismatches (as SAMtools). The coefficient for downgrading mapping quality for reads containing excessive mismatches. Given a read with a phred-scaled probability q of being generated from the mapped position, the new mapping quality is about sqrt((INT-q)/INT)*INT. A zero value disables this functionality; if enabled, the recommended value for BWA is 50;

- Baq=1: BAQ is the Phred-scaled probability of a read base being misaligned. It greatly helps to reduce false SNPs caused by misalignments. BAQ is calculated using the probabilistic realignment method described in the paper "Improving SNP discovery by base alignment quality", Heng Li, Bioinformatics, Volume 27, Issue 8 ;
- uniqueOnly=1: remove reads that have multiple best hits. 0 no (default), 1 remove;
- remove_bads=1: same as the samtools flags -x which removes read with a flag above 255 (not primary, failure and duplicate reads). 0 no 1 remove (default).

Haplotype calling

- doGeno=4: print the called genotype as AA, AC, AG;

  =32: write the posterior probabilities of the 3 genotypes as binary;

- doPost=1: estimate the posterior genotype probability based on the allele frequency as a prior;
- GL=2: Genotype likelihoods obtained by using GATK algorithm
- postCutoff=0.99: call only a genotype with a posterior above this threshold;
- minInd=28: only keep sites with at least minIndDepth (default is 1) from at least [int] individuals
- -doMaf=2: Here the major allele is assumed to be known (inferred or given) however the minor allele is not determined. Instead, we sum over the 3 possible minor alleles weighted by their probabilities;
- SNP_pval= 1e-3: only work with sites with a p-value less than 1e-3;
- -doGlf=2: beagle haplotype imputation and be performed directly on genotype likelihoods. To generate beagle input file use;
- doMajorMinor=1: from the input for either sequencing data like bam files or from genotype likelihood data like. The major and minor allele can be inferred directly from likelihoods;
- doCounts=1: use -doCounts 1 to count the bases at each site after filters.

Individual clustering and population structure parameters used in ANGSD

Principal Component Analysis (PCA) for the whole genomes

uniqueOnly 1, remove_bads 1, only_proper_pairs 1, C 50, baq 1, setMinDepth 30, setMaxDepth 600, setMinDepthInd 5, setMaxDepthInd 100, doCounts 1, GL 2, doMajorMinor 1, doMaf 1, SNP_pval 1e-3, doGeno 32, doPost 1, nThreads 10, doPlink 2

Principal Component Analysis (PCA) for the ddRAD-seq

uniqueOnly 1, remove_bads 1, only_proper_pairs 0, C 50, baq 1, setMinDepth 5, setMaxDepth 235, setMinDepthInd 5, setMaxDepthInd 4700, doCounts 1, GL 2, doMajorMinor 1, doMaf 1, SNP_pval 1e-3, doGeno 32, doPost 1, -nThreads 10, doPlink 2

Admixture and F_{ST} for ddRAD-seq

doPlink 2, uniqueOnly 1, remove_bads 1, only_proper_pairs 0, C 50, baq 1, setMinDepth 235, setMaxDepth 4700, setMinDepthInd 5, setMaxDepthInd 100, doCounts 1, GL 2, doMajorMinor 1, doGlf 2, SNP_pval 1e-3, minInd 28, doGeno 4, doPost 1, doMaf 2, postCutoff 0.99, geno_minDepth 4

The option -*doSaf 1* was applied with the parameters above described to generate the Site Frequency Spectrum and to estimate the weighted F_{ST} population differentiation index.

Hybrid Detection with HyDe program

Commands used were:

run_hyde.py -i Aaustralis_clade_variants_thinned25kb.phy -m Paper2-map.txt -o New_Zealand -n 6 -t 4 -s 97047

individual_hyde.py -i Aaustralis_clade_variants_thinned25kb.phy -m Paper2-map.txt -tr inds.txt -o New_Zealand -n 6 -t 4 -s 97047

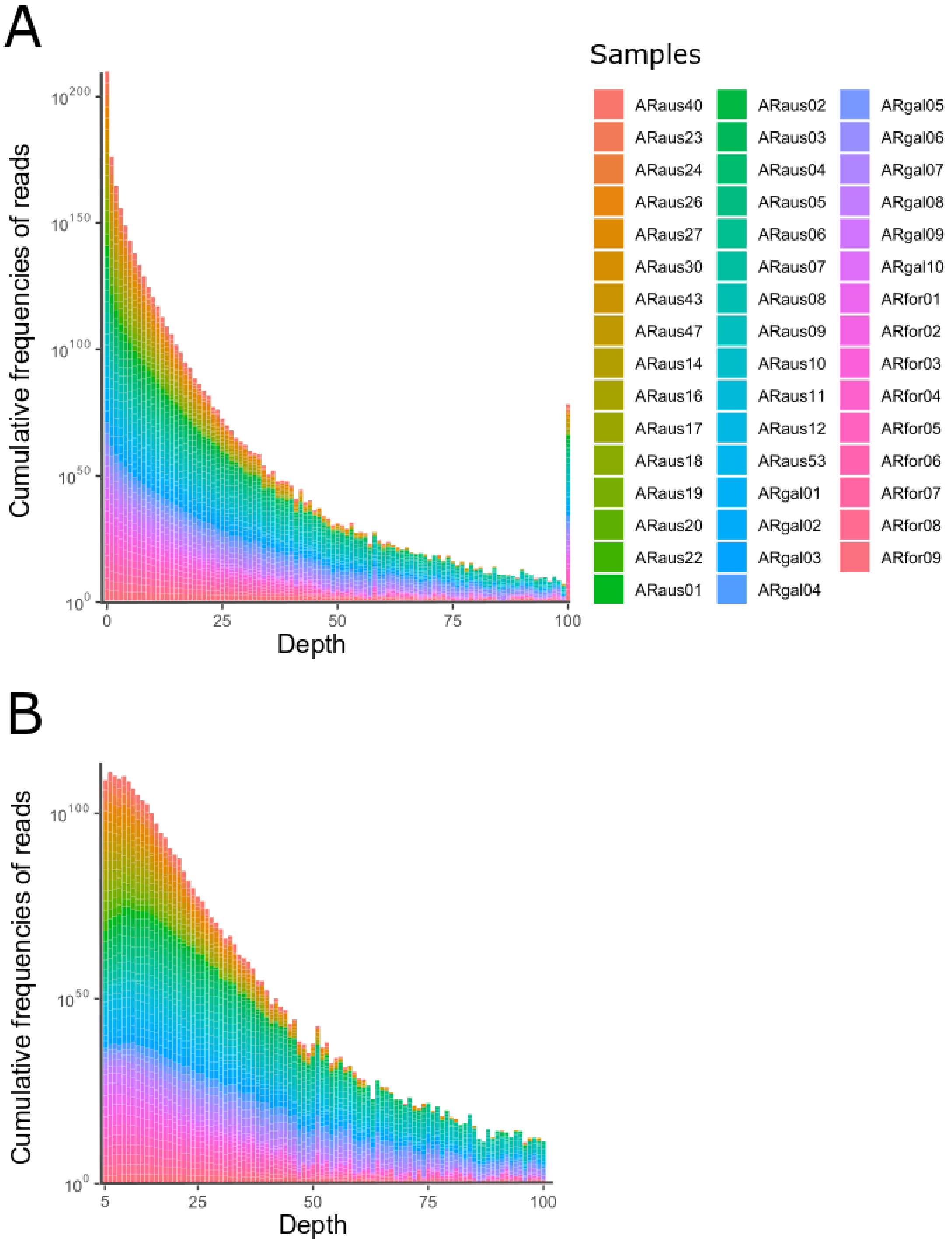

Fig. S1.
Cumulative frequencies of reads depth of the ddRAD-seq dataset. (A) before and (B) after SNP filtering.

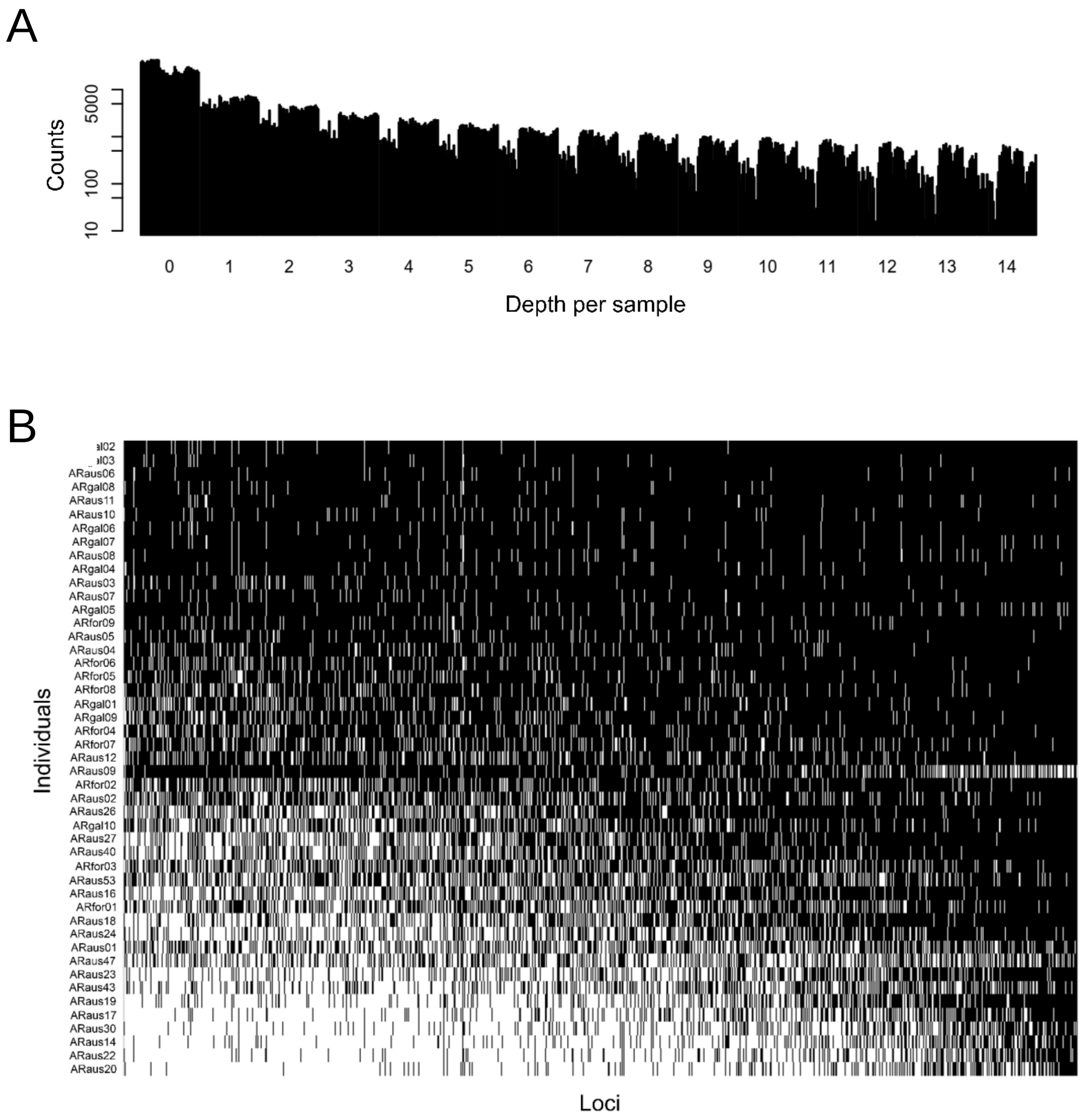

Fig. S2.
Effect of data filtering on ddRAD-seq data. (A) ddRAD-seq pre-filtering depth per sample. The chart was pruned to show reads removed after the minimum individual depth per sample (<5). The x-axis shows the total depth until 14X; the y-axis shows the counts of reads to each sample (black bar) and its respective depth. (B) Matrix of the ddRAD-seq SNP dataset represents the 3,198 SNPs. The graphic shows the presence (black) and absence (white) of genomic information by loci and sample. The individuals were sorted by the amount of data (higher to lower).

A

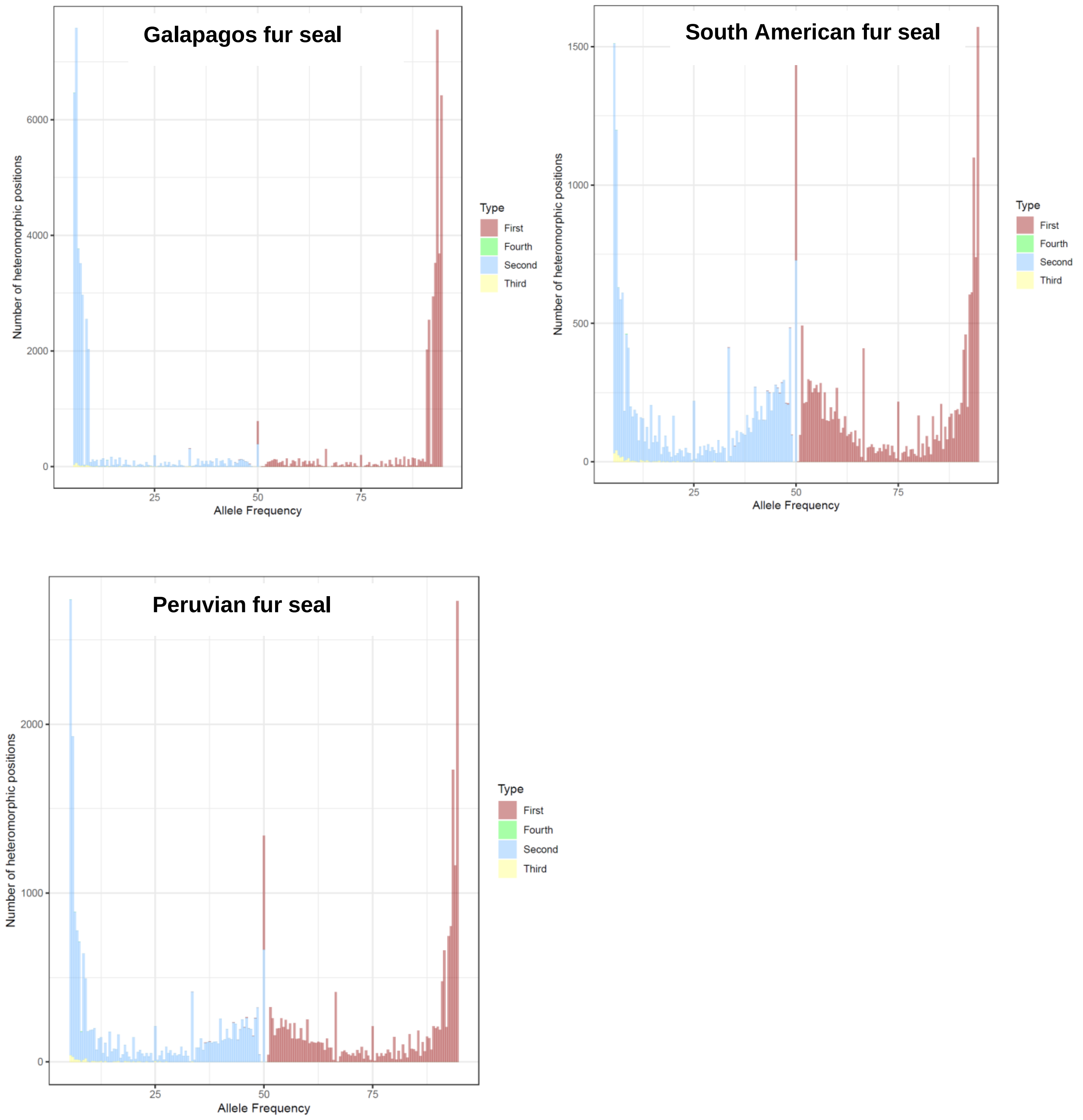
Galapagos fur seal
South American fur seal
Peruvian fur seal
Number of heteromorphic positions
Allele Frequency
0
2000
4000
6000
500
1000
1500
25
50
75
Type
First
Fourth
Second
Third

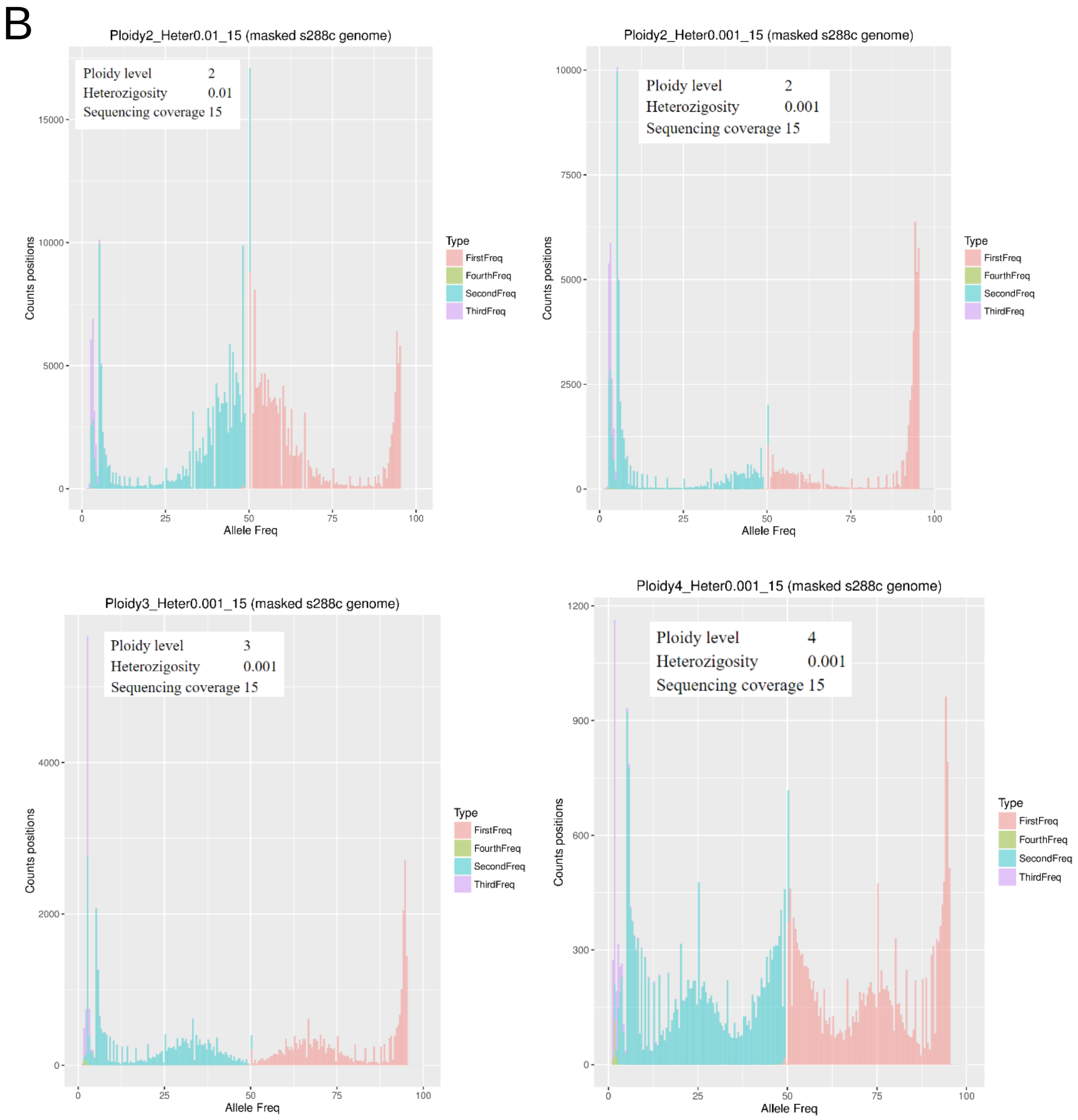
B
Ploidy2_Heter0.01_15 (masked s288c genome)
Ploidy level 2
Heterozigosity 0.01
Sequencing coverage 15
Counts positions
Allele Freq
Type
FirstFreq
FourthFreq
SecondFreq
ThirdFreq
Ploidy2_Heter0.001_15 (masked s288c genome)
Ploidy level 2
Heterozigosity 0.001
Sequencing coverage 15
Ploidy3_Heter0.001_15 (masked s288c genome)
Ploidy level 3
Heterozigosity 0.001
Sequencing coverage 15
Ploidy4_Heter0.001_15 (masked s288c genome)
Ploidy level 4
Heterozigosity 0.001
Sequencing coverage 15

C

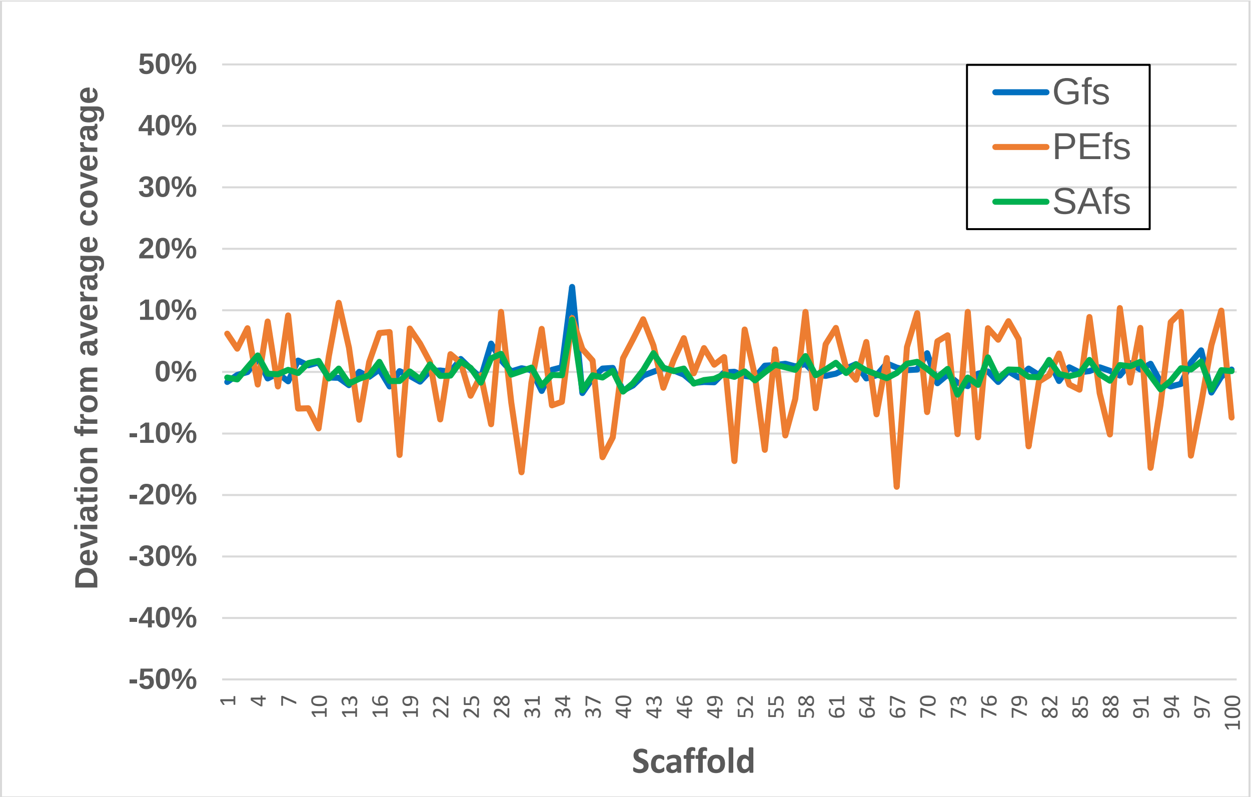

Fig. S3.

Evaluation of chromosome changes in the studied species. Results of the *ploidyNGS* script. (A) Empirical results estimated on the genomes of our three focal species. All three individuals are more similar to diploidy. (B) Expected (simulated) results in four situations (from https://goo.gl/BHHNYM). Ploidy, heterozygosity, and coverage as indicated. NOTE: The expected frequencies are, for a diploid: 0, 50, 100; for a triploid: 0, 33, 66, 100; and for a tetraploid: 0, 25, 5, 75, 100. Reduced heterozygosity and coverage reduce the peaks of the non-fixed states (not 0 or 100). (C) Test for aneuploidy. Deviation from the average genome coverage for the 100 largest scaffolds. No scaffold of any species varied more than 20%. The expected variation for a trisomy and a tetrasomy is 50% and 100% (increase or decrease from the genome average), respectively.

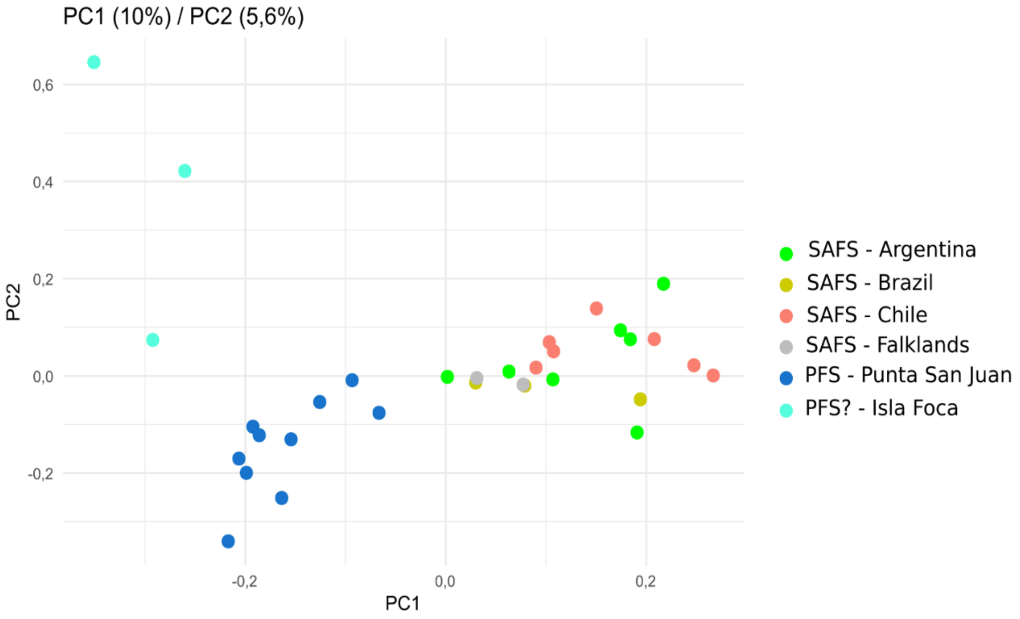

Fig. S4.
PCA using ddRAD-seq SNPs. PCA only the SAfs and Pfs samples. Isla Foca samples, initially assigned as Peruvian fur seals, are represented with light blue dots and with an interrogation signal in the legend.

A

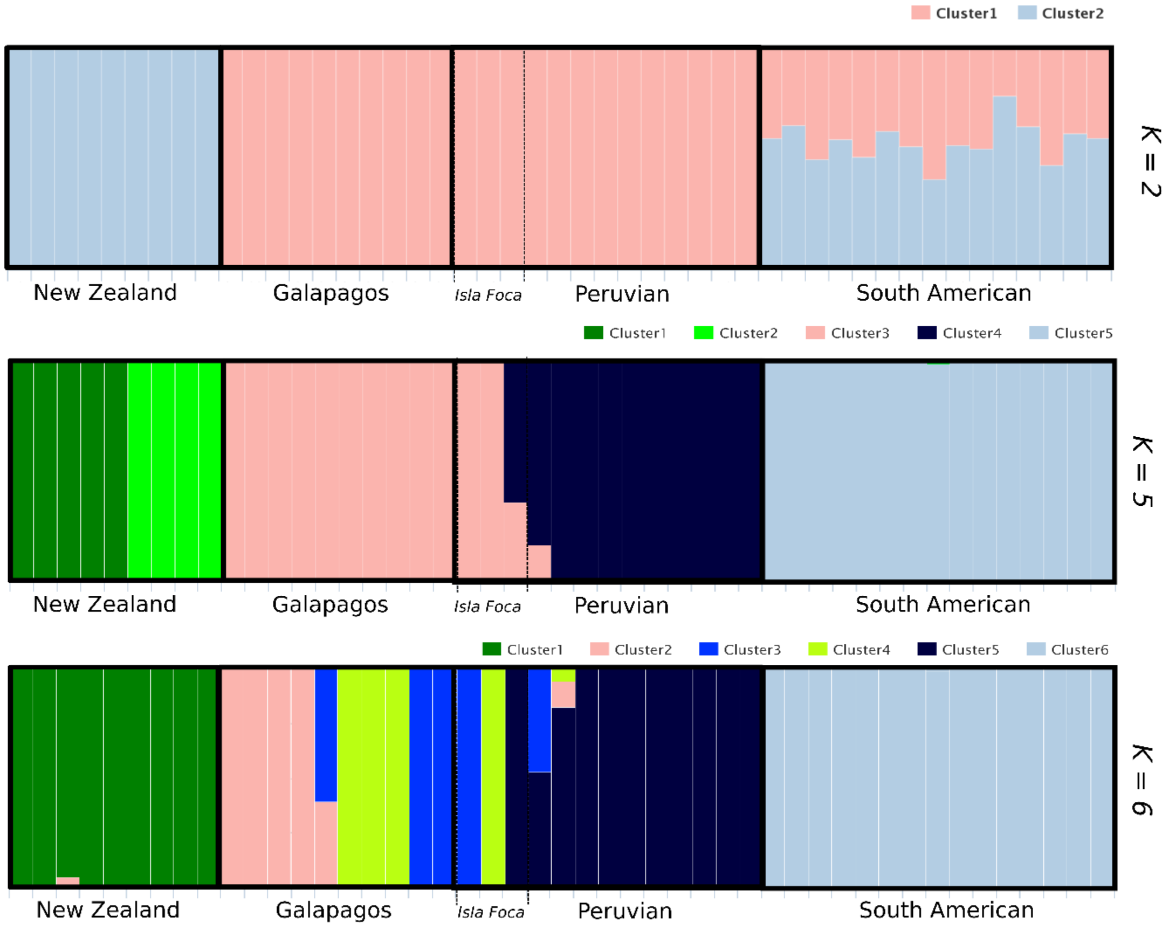

B

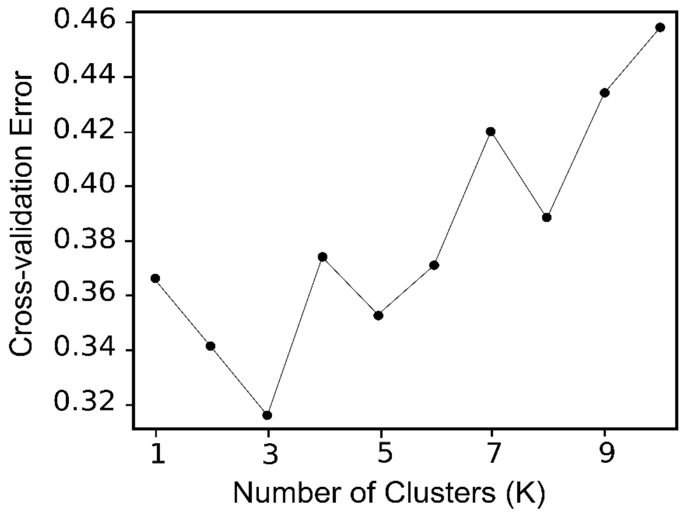

Fig. S5.

Exploratory admixture analyses. (A) Admixture plots based on ddRAD-seq data with K=2, K=5, and K=6. (B) Cross-validation chart showing K=3 with lowest error rate calculated in Admixture (from 1 to 10 clusters - Alexander and Lange 2011).

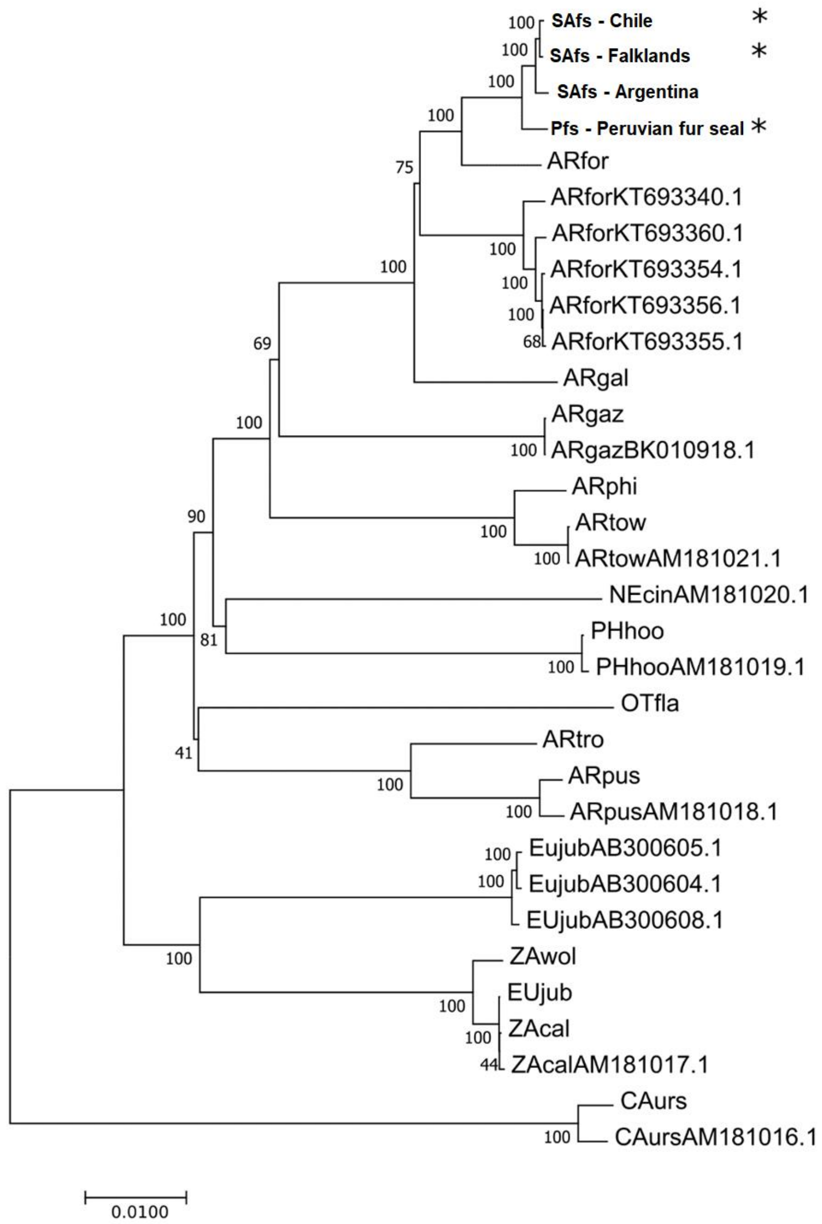

Fig. S6.
Neighbor-joining (NJ) cladogram inferred from mitogenomes. NJ tree was recovered in MEGA-X using p-distance, complete deletion, and 500 bootstrap replicates. The asterisks represent the *Arctocephalus australis* mitochondrial DNA genomes sequenced in this study from Peru (Peruvian fur seal - Pfs), Argentina, Falklands/Malvinas, and Chile (SAfs -). The other sequences were otariids from Lopes et al. (*18*) (labels without numbers, e.g., ARfor, ARgal, etc.) or from GenBank (whose labels included their accession numbers). ARfor = *A. forsteri*, NZfs, ARgal = *A. galapagoensis,* Gfs, and ARgaz = *A. gazella*, Afs.

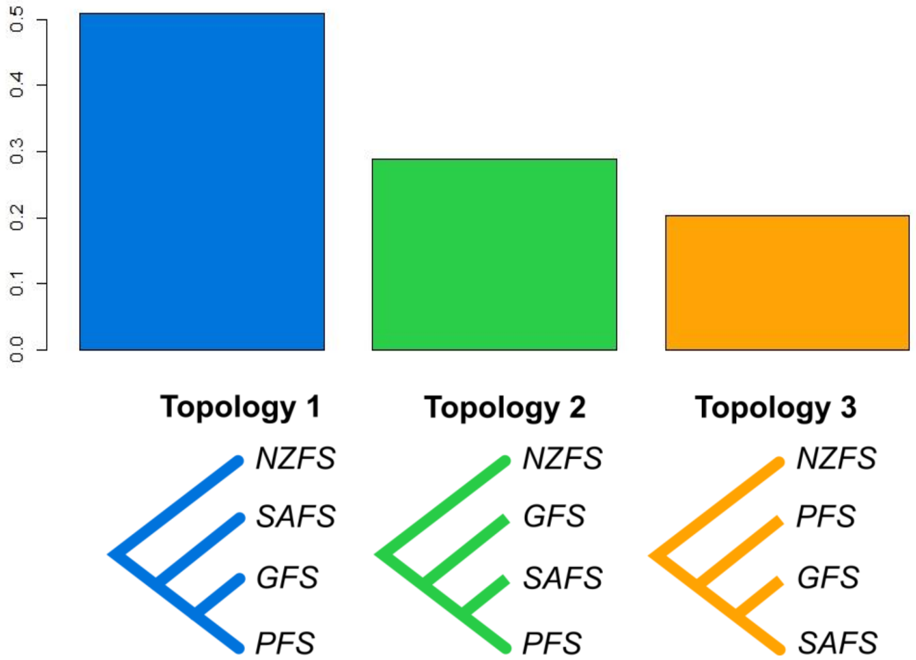

Fig. S7.

Topology estimation across genomes. Relative frequency (top) of the three main topologies (bottom) over 3,983 genomic windows from the 21 largest scaffolds.

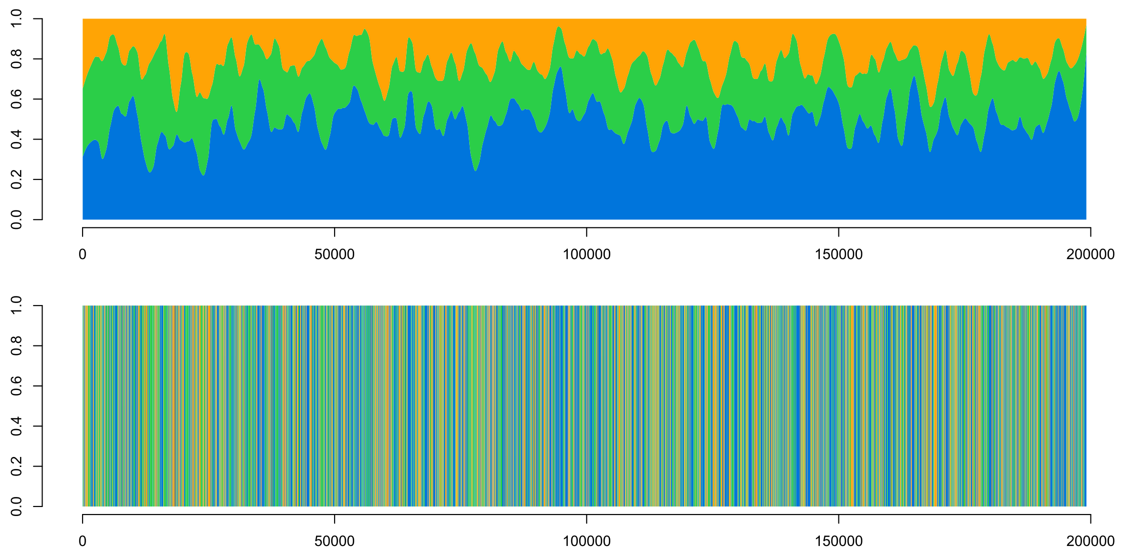

Fig. S8.

The phylogenetic discordance along the 21 largest scaffolds. The analysis comprised 3,983 trees and approximately 200 Mb. Top, with smoothing (0.03), bottom (no smoothing).

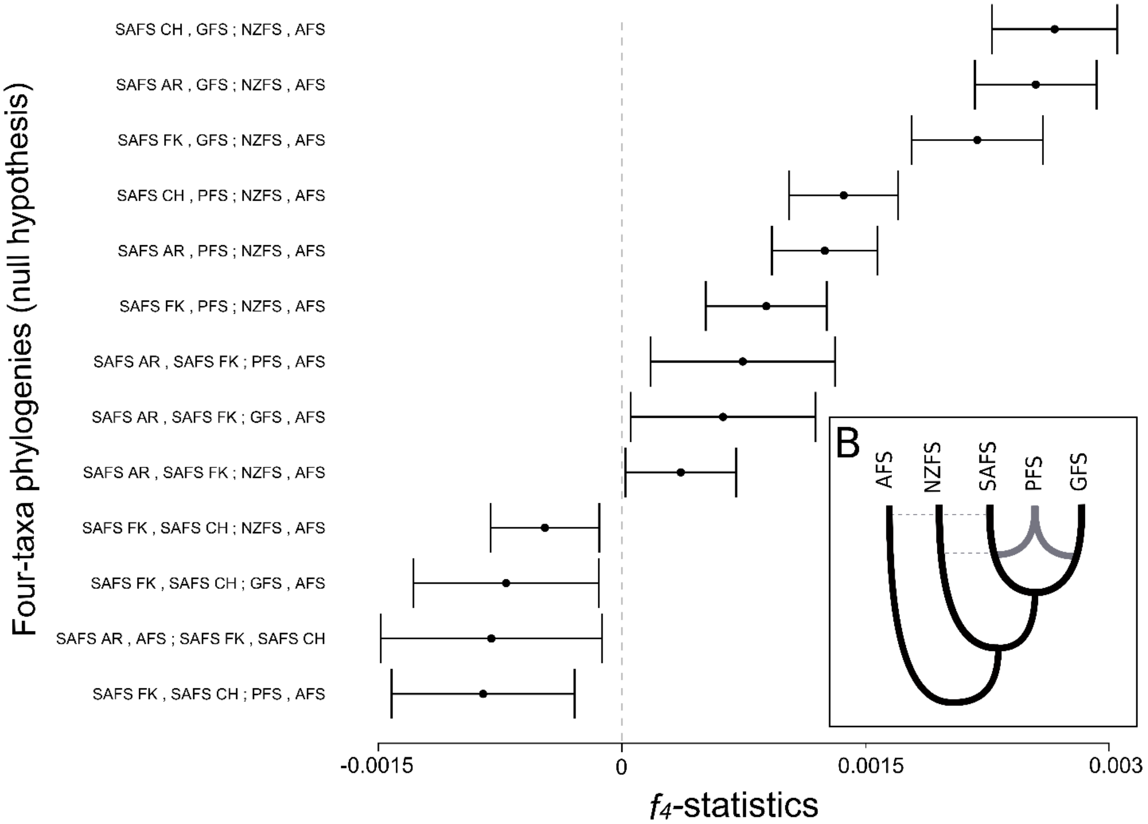

Fig. S9.
Estimation of ancestry proportions in admixed populations using *F*$_4$-statistics. (A) Proposed four-taxa phylogenies and f_4-statistics distribution showing the evidence for genomic introgression between New Zealand fur seal (NZFS)/Antarctic fur seal (AFS) and South American fur seal. South American fur seal: SAFS AR - Argentina, FK - Falkland/Malvinas Islands and CH - Chile. All f_4 results were significant |Z-score| > 3. (B) Migration events represented in the network from NZFS to all SAFS and PFS, and from AFS to SAFS from Falkland/Malvinas Islands.

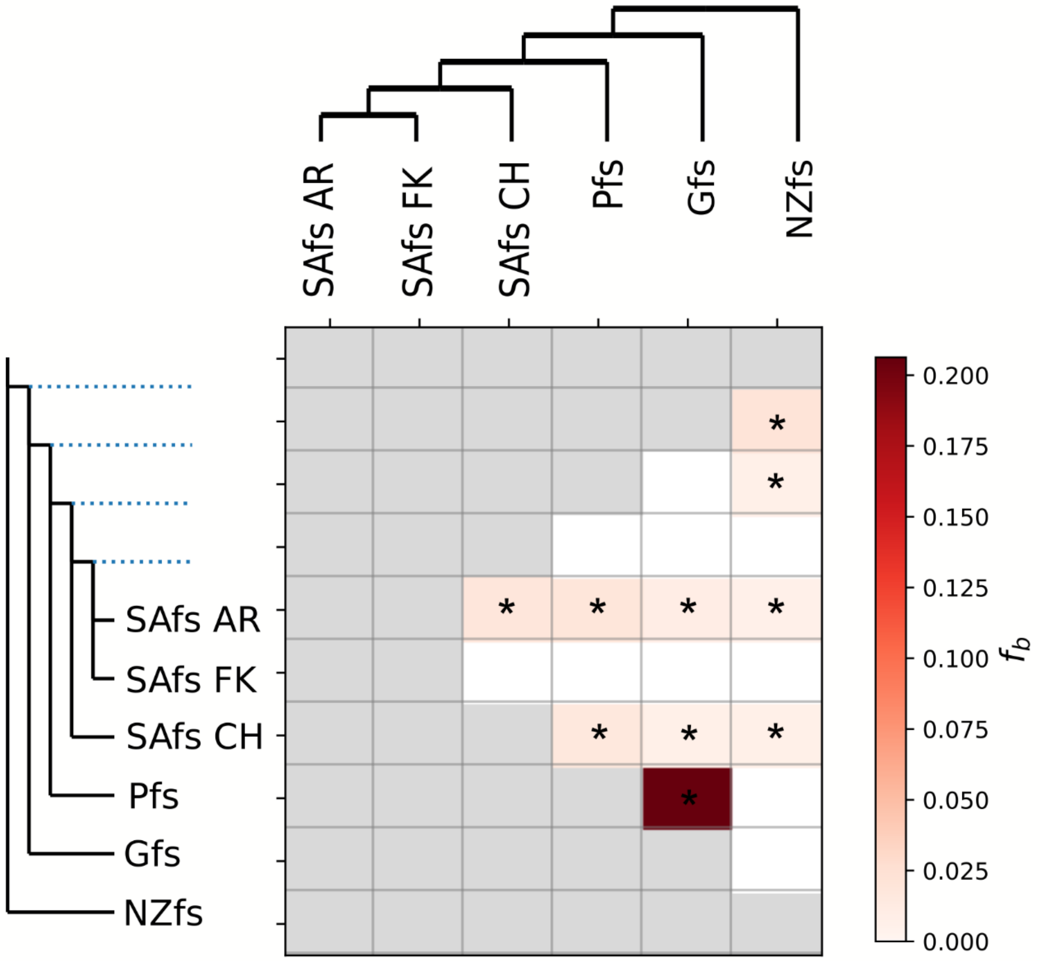

Fig. S10.
Estimation of ancestry proportions in admixed populations using *F*-branch. *F*-branch results showing the excess sharing of alleles between branches. The scenario shows that Peruvian fur seals (PFS) underwent strong event of genomic introgression. The x-axis is the proposed topology and the y-axis, the topology with ancestral taxa represented by dashed lines. The different sampled localities of SAFS are represented as follows: Argentina (AR), Falklands/Malvinas (FK), and Chile (CH). New Zealand fur seals (NZFS) is the basal taxon. The red color gradient in the right side of the figure represents the intensity of introgression of the metric (f_b(C)) and the asterisks indicate significant results $P > 0.01$.

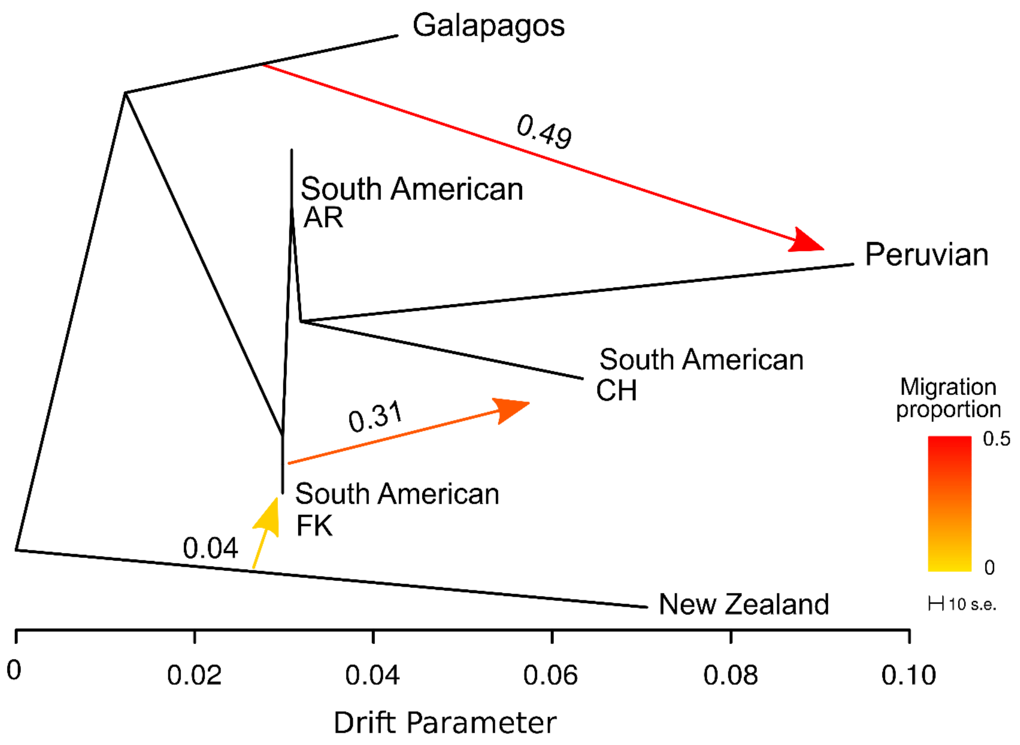

Fig. S11.
Estimation of unidirectional genomic introgression. Maximum-Likelihood phylogeny of TreeMix showing Peruvian fur seal grouping with South American fur seals and a strong signal of introgression incoming from Galapagos fur seals. South American fur seals: Argentina (AR), Falklands/Malvinas (FK) and Chile (CH).

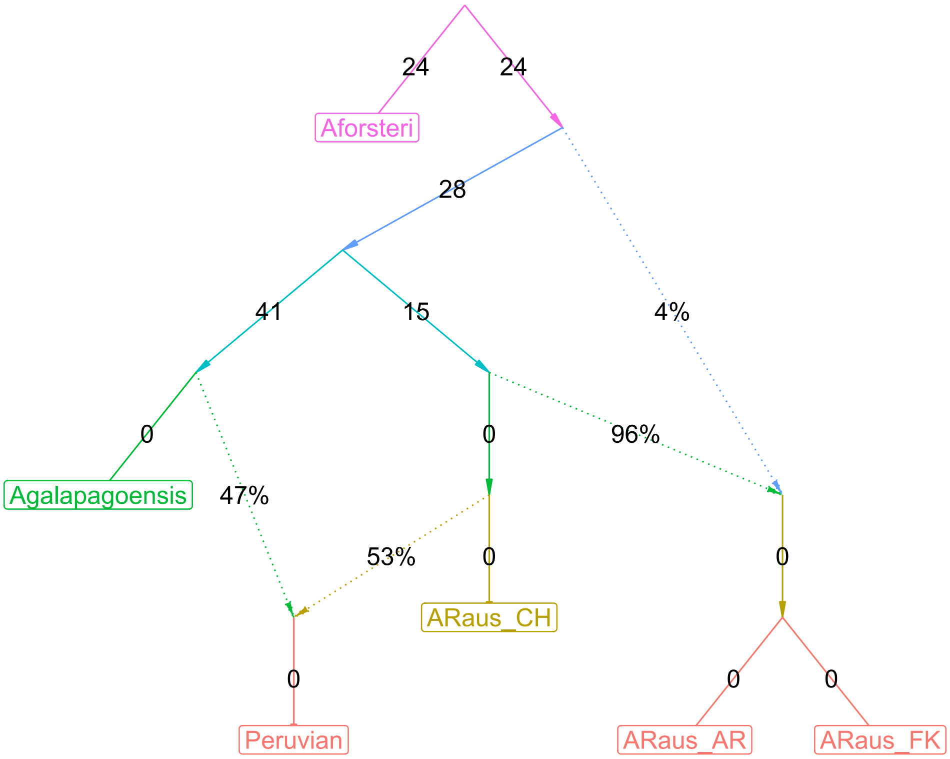

Fig. S12.

Inferred admixture graph with admixtools2 with the three SAfs populations separated. The numbers next to branches represent the percentage of ancestry from each parental node.

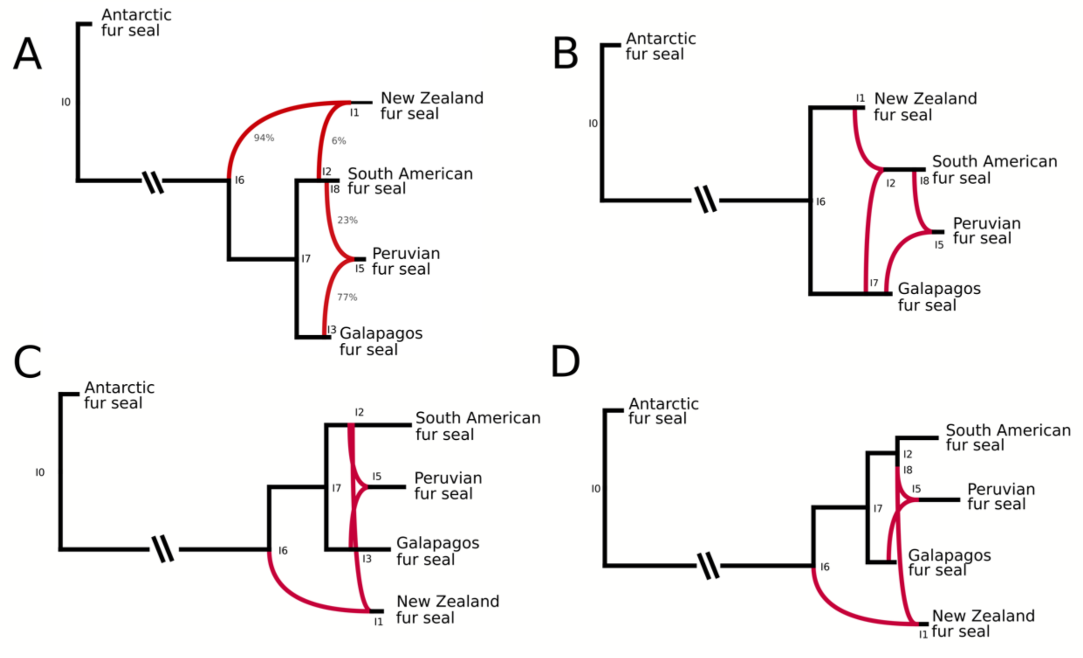

Fig. S13.

Potential alternative scenarios of evolution estimated by Phylonet. The rooted networks with the highest Maximum Posterior (MAP) value within the 95% credible set of topologies inferred by PhyloNet and 7,810 gene trees. (A) 55.9%, (B) 22,1, (C)15.4%, and (D) 5.4%. The networks show the hybrid origin of the Peruvian fur seals from introgressive events between Galapagos and South American fur seals and past hybridization between South American and New Zealand fur seals. The percentage in (A) represents the amount of introgression, and the branch lengths are in coalescent units.

A

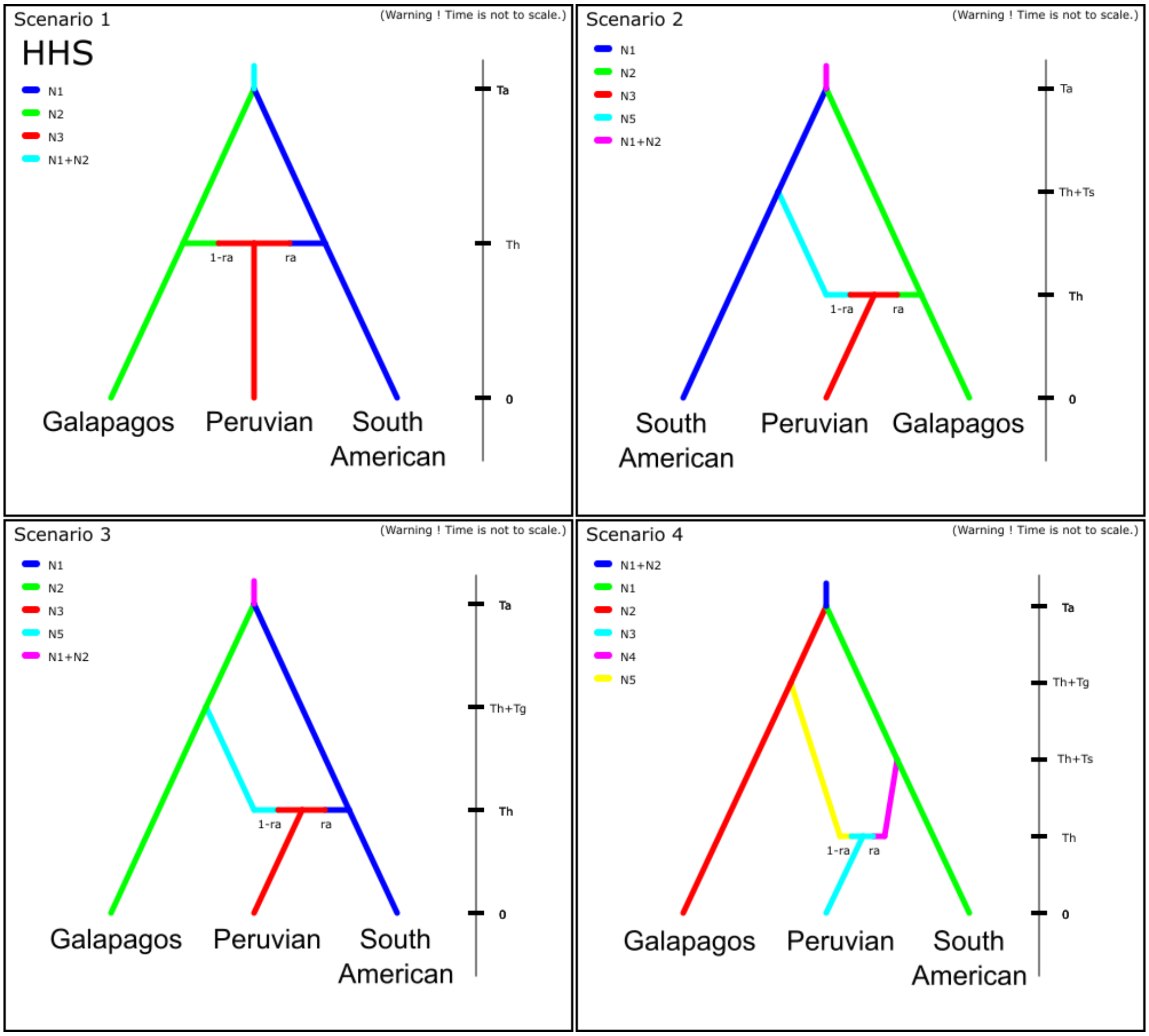

Scenario 1
(Warning ! Time is not to scale.)
HHS
N1
N2
N3
N1+N2
1-ra
ra
Ta
Th
0
Galapagos
Peruvian
South American
Scenario 2
(Warning ! Time is not to scale.)
N1
N2
N3
N5
N1+N2
Ta
Th+Ts
Th
0
1-ra
ra
South American
Peruvian
Galapagos
Scenario 3
(Warning ! Time is not to scale.)
N1
N2
N3
N5
N1+N2
Ta
Th+Tg
Th
0
1-ra
ra
Galapagos
Peruvian
South American
Scenario 4
(Warning ! Time is not to scale.)
N1+N2
N1
N2
N3
N4
N5
Ta
Th+Tg
Th+Ts
Th
0
1-ra
ra
Galapagos
Peruvian
South American

B

SCENARIO 1
N1 N2 N3
0 sample 1
0 sample 2
0 sample 3
Th split 3 1 2 ra
Ta merge 1 2
Ta VarNe 1 N1+N2

SCENARIO 2
N1 N2 N3 N2 N5
0 sample 1
0 sample 2
0 sample 3
Th split 3 4 5 ra
Th merge 2 4
Th+Ts merge 1 5
Ta merge 1 2
Ta VarNe 1 N1+N2

SCENARIO 3
N1 N2 N3 N1 N5
0 sample 1
0 sample 2
0 sample 3
Th split 3 4 5 ra
Th merge 1 4
Th+Ts merge 2 5
Ta merge 1 2
Ta VarNe 1 N1+N2

SCENARIO 4
N1 N2 N3 N4 N5
0 sample 1
0 sample 2
0 sample 3
Th split 3 4 5 ra
Th+Ts merge 1 4
Th+Tg merge 2 5
Ta merge 1 2
Ta VarNe 1 N1+N2

C

Scenario	○ Uniform ◉ Other	scenario 1	scenario 2	scenario 3	scenario 4
		0.25	0.25	0.25	0.25

parameters		Uniform	Log-uniform	Normal	Log-normal	minimum	maximum
N1		◉	○	○	○	1000	5000000
N2	set condition	◉	○	○	○	1000	1000000
N3	set condition	◉	○	○	○	1000	1000000
Th		◉	○	○	○	10.0	10000.0
ra		◉	○	○	○	0.01	0.999
Ta	set condition	◉	○	○	○	10.0	10000.0
N5	set condition	◉	○	○	○	10.0	10000
Ts	set condition	◉	○	○	○	10.0	10000.0
Tg	set condition	◉	○	○	○	10.0	10000.0
N4	set condition	◉	○	○	○	10.0	10000

Fig. S14.
ABC analysis in DIYABC. (A) Tested scenarios as depicted in DIYABC. Scenario 1 is an HHS, scenarios 2 and 3 represent introgression from Gfs into a previously isolated population of SAfs originating the Pfs or from SAfs into a previously isolated population of Gfs originating the Pfs (respectively), scenario 4 represents the hybrid origin of Pfs from the fusion of two previously isolated populations from Gfs and SAfs. (B) Scenarios specifications. (C) Parameters prior distributions.

A

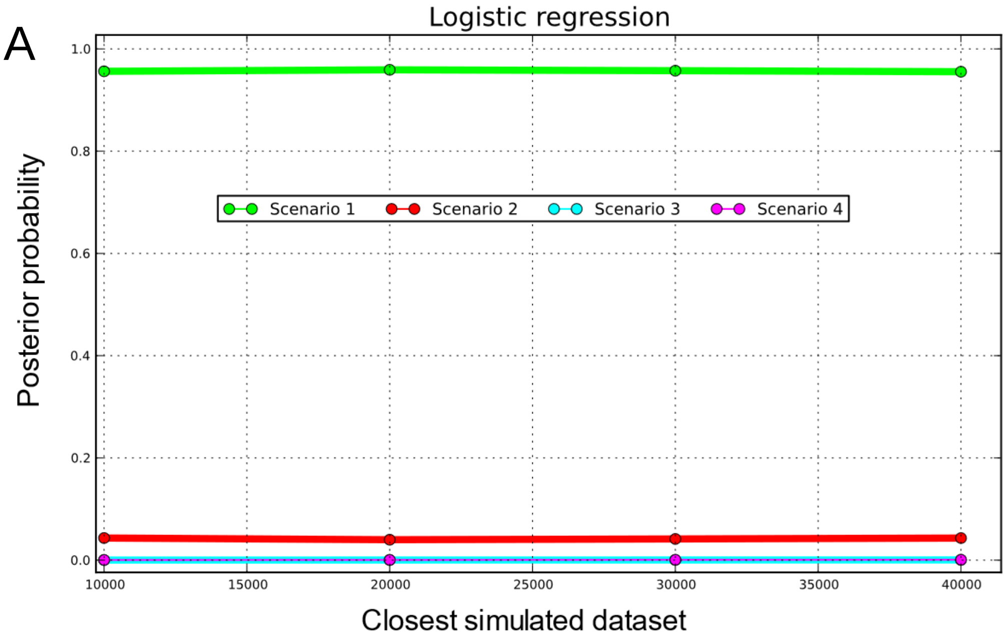

B

COMPARISON OF SCENARIOS

Candidate scenarios : [1, 2, 3, 4]

Number of simulated data sets : 4000000

Logistic approach

n	scenario 1	scenario 2	scenario 3	scenario 4
10000	0.9562 [0.9416,0.9707]	0.0433 [0.0000,0.3609]	0.0002 [0.0000,0.3177]	0.0004 [0.0000,0.3179]
20000	0.9591 [0.9497,0.9686]	0.0400 [0.0000,0.2619]	0.0003 [0.0000,0.2221]	0.0005 [0.0000,0.2223]
30000	0.9574 [0.9495,0.9653]	0.0415 [0.0000,0.2194]	0.0004 [0.0000,0.1783]	0.0007 [0.0000,0.1786]
40000	0.9554 [0.9483,0.9625]	0.0432 [0.0000,0.1950]	0.0005 [0.0000,0.1522]	0.0008 [0.0000,0.1525]

C

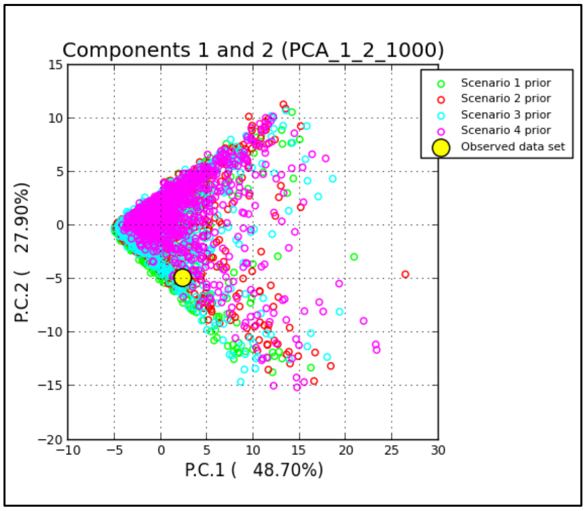

Fig. S15.

Posterior parameter distribution of ABC analysis. (A) Posterior probability of each scenario (scenario choice) computed with logistic regression. The posterior probability of each scenario (y-axis) was computed with the logistic regression based on the specified number of the closest simulated datasets (x-axis). Scenarios 3 and 4 values are close to zero and superposed. (B) Numerical values for scenario choice posterior probabilities (seen in A). n=number of the closest simulated datasets. (C) PCA pre-evaluated scenario prior

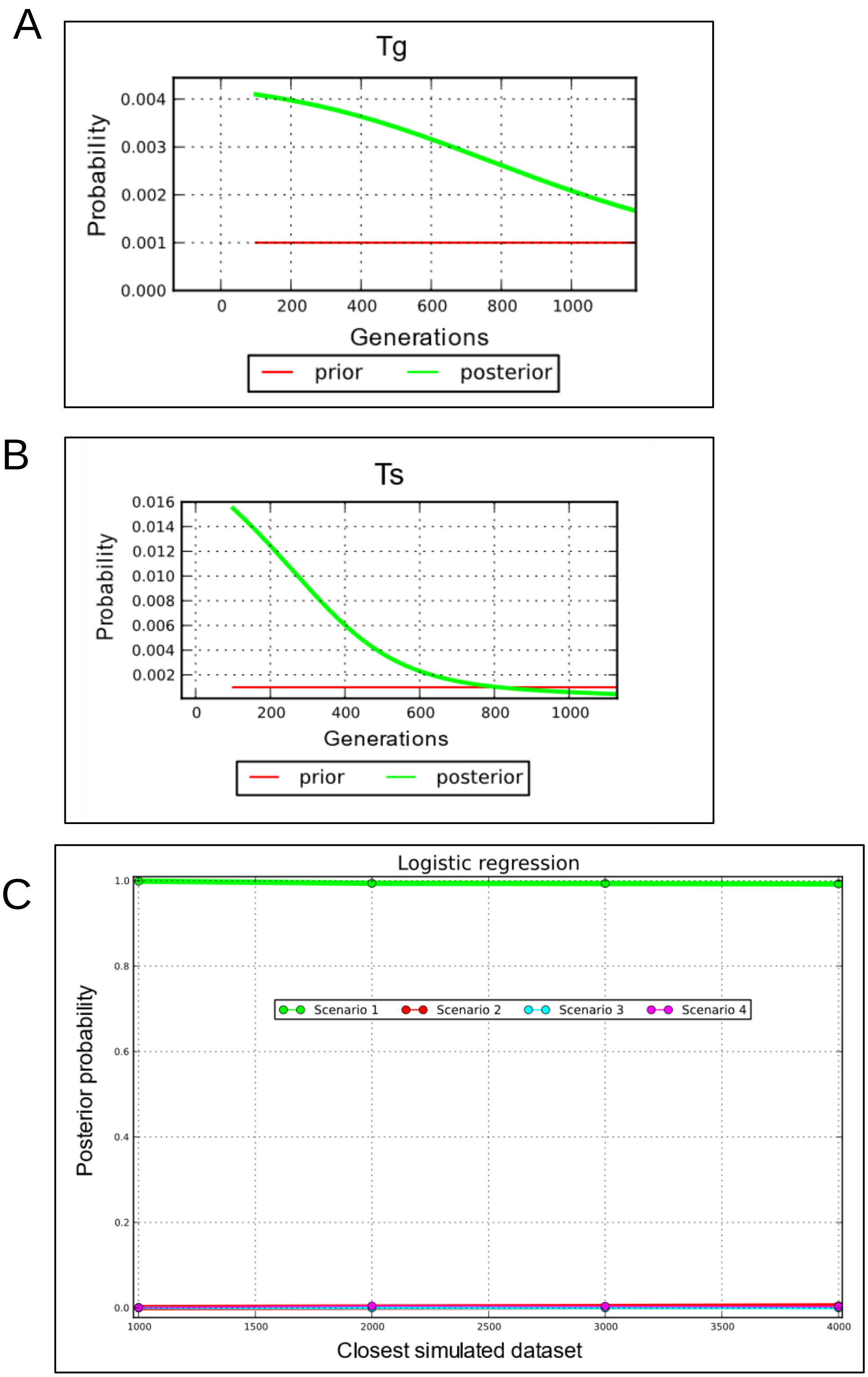

Fig. S16.
Posterior distributions of parameters Tg (A) and Ts (B) in scenarios 2-4 showing that they approached the minimum possible value of 100 generations. (**C**) Scenario choice with logistic regression when **Tg** and **Ts** priors are set a minimum of 300 generations instead of 100 generations as in fig. S15. HHS scenario 1 approached a posterior probability of 1. Axes are the same as in Fig. S15A. Values for scenarios 2-4 are close to zero and superposed.

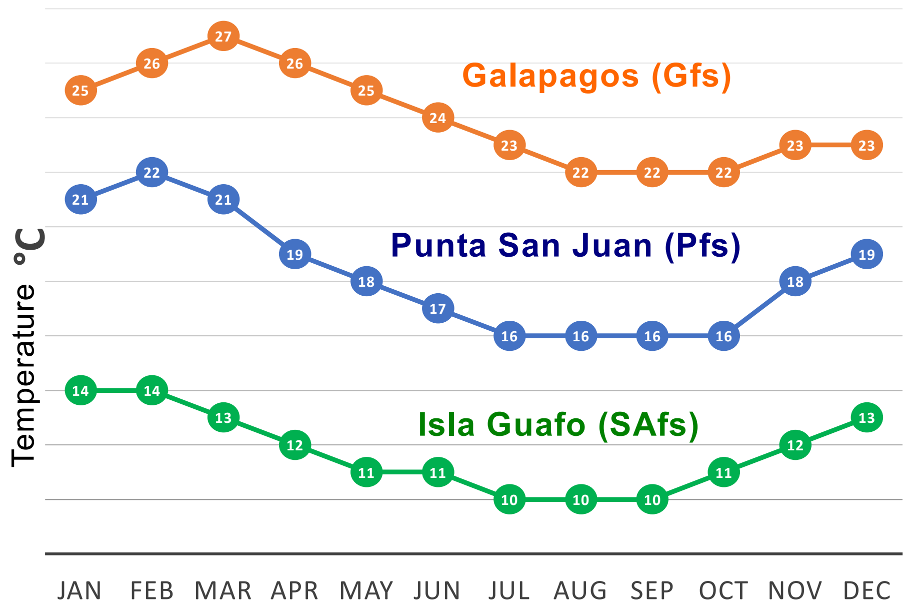

Fig. S17.
Sea surface temperature monthly average in the main area of occurrence of the three species. Isla Guafo (Chile) is the northernmost breeding colony of the SAfs *s. str*. Data obtained from MODIS AQUA satellite. Doi: 10.5067/VJAFPLI1CSIV.

Table S1.
PALEOMIX statistics for each species mapped against the *O. rosmarus* genome (2.4 Gb genome). The asterisks represent whole genomes used in Lopes et al. (*18*). SAfs - South American fur seal; Pfs - Peruvian fur seal; Gfs - Galapagos fur seal; NZfs - New Zealand fur seal; Afs - Antarctic fur seal.

	Library source	GenBank Access nº	Coverage	Proportion Ref. Genome Mapped	Fraction of Mapped reads retained	Fraction of Unmapped reads	Fraction of PCR duplicates
SAfs - Argentina*	GenBank	SRX6989525	21.34	0.85	0.91	0.09	0.06
SAfs - Falklands/Malvinas	TruSeq Nano	SRX17089589	17.39	0.85	0.89	0.11	0.04
SAfs - Chile	TruSeq Nano	SRX17089588	32.04	0.81	0.90	0.10	0.10
Pfs	TruSeq PCR Free	SRX17062720	27.91	0.82	0.92	0.80	0.11
Gfs*	GenBank	SRX7011050	15.42	0.79	0.90	0.10	0.12
NZfs*	GenBank	SRX7011168	26.05	0.86	0.91	0.09	0.05
Afs	GenBank	SRX1338463-82	32.44	0.83	0.89	0.11	0.07

Table S2.
Sampling information for fur seals samples used in ddRAD-seq analyses.

Species	Common name	ID code	Locality	Country	Geographical coordinates	Filtered out
Arctocephalus australis	Peruvian fur seal	Aaus01	Punta San Juan	Peru	15°22’S, 75°11’W	No
Arctocephalus australis	Peruvian fur seal	Aaus02	Punta San Juan	Peru	15°22’S, 75°11’W	No
Arctocephalus australis	Peruvian fur seal	Aaus03	Punta San Juan	Peru	15°22’S, 75°11’W	No
Arctocephalus australis	Peruvian fur seal	Aaus04	Punta San Juan	Peru	15°22’S, 75°11’W	No
Arctocephalus australis	Peruvian fur seal	Aaus05	Punta San Juan	Peru	15°22’S, 75°11’W	No
Arctocephalus australis	Peruvian fur seal	Aaus06	Punta San Juan	Peru	15°22’S, 75°11’W	No
Arctocephalus australis	Peruvian fur seal	Aaus07	Punta San Juan	Peru	15°22’S, 75°11’W	No
Arctocephalus australis	Peruvian fur seal	Aaus08	Punta San Juan	Peru	15°22’S, 75°11’W	No
Arctocephalus australis	Peruvian fur seal	Aaus09	Punta San Juan	Peru	15°22’S, 75°11’W	No
Arctocephalus australis	Peruvian fur seal	Aaus12	Punta San Juan	Peru	15°22’S, 75°11’W	No
Arctocephalus australis	Peruvian fur seal	Aaus10	Isla Foca	Peru	05°12’S, 81°12’W	No
Arctocephalus australis	Peruvian fur seal	Aaus11	Isla Foca	Peru	05°12’S, 81°12’W	No
Arctocephalus australis	Peruvian fur seal	Aaus53	Isla Foca	Peru	05°12’S, 81°12’W	No
Arctocephalus australis	South American fur seal	Aaus13	Southern Chile	Chile	36°36’S, 73°11’W	Yes
Arctocephalus australis	South American fur seal	Aaus14	Southern Chile	Chile	36°36’S, 73°07’W	No
Arctocephalus australis	South American fur seal	Aaus15	Southern Chile	Chile	36°36’S, 73°07’W	Yes
Arctocephalus australis	South American fur seal	Aaus16	Southern Chile	Chile	36°36’S, 73°07’W	No
Arctocephalus australis	South American fur seal	Aaus17	Southern Chile	Chile	36°36’S, 73°07’W	No
Arctocephalus australis	South American fur seal	Aaus18	Southern Chile	Chile	36°36’S, 73°07’W	No
Arctocephalus australis	South American fur seal	Aaus19	Southern Chile	Chile	36°36’S, 73°07’W	No
Arctocephalus australis	South American fur seal	Aaus20	Southern Chile	Chile	36°36’S, 73°07’W	No
Arctocephalus australis	South American fur seal	Aaus21	Southern Chile	Chile	36°36’S, 73°07’W	Yes
Arctocephalus australis	South American fur seal	Aaus22	Southern Chile	Chile	36°36’S, 73°07’W	No
Arctocephalus australis	South American fur seal	Aaus23	Province of Chubut	Argentina	45°06’S, 65°24’W	No
Arctocephalus australis	South American fur seal	Aaus24	Province of Chubut	Argentina	45°06’S, 65°24’W	No
Arctocephalus australis	South American fur seal	Aaus25	Province of Chubut	Argentina	45°06’S, 65°24’W	Yes
Arctocephalus australis	South American fur seal	Aaus26	Province of Chubut	Argentina	45°06’S, 65°24’W	No
Arctocephalus australis	South American fur seal	Aaus27	Province of Chubut	Argentina	45°06’S, 65°24’W	No
Arctocephalus australis	South American fur seal	Aaus28	Province of Chubut	Argentina	45°06’S, 65°24’W	Yes
Arctocephalus australis	South American fur seal	Aaus29	Province of Chubut	Argentina	45°06’S, 65°24’W	Yes
Arctocephalus australis	South American fur seal	Aaus30	Province of Chubut	Argentina	45°06’S, 65°24’W	No
Arctocephalus australis	South American fur seal	Aaus31	Province of Chubut	Argentina	45°06’S, 65°24’W	Yes
Arctocephalus australis	South American fur seal	Aaus32	Province of Chubut	Argentina	45°06’S, 65°24’W	Yes
Arctocephalus australis	South American fur seal	Aaus33	Rio Grande do Sul	Brazil	29°20’S, 49°’42’W	Yes
Arctocephalus australis	South American fur seal	Aaus34	Rio Grande do Sul	Brazil	29°20’S, 49°’42’W	Yes
Arctocephalus australis	South American fur seal	Aaus35	Rio Grande do Sul	Brazil	29°20’S, 49°’42’W	Yes
Arctocephalus australis	South American fur seal	Aaus36	Rio Grande do Sul	Brazil	29°20’S, 49°’42’W	Yes
Arctocephalus australis	South American fur seal	Aaus37	Rio Grande do Sul	Brazil	29°20’S, 49°’42’W	Yes
Arctocephalus australis	South American fur seal	Aaus38	Rio Grande do Sul	Brazil	29°20’S, 49°’42’W	Yes
Arctocephalus australis	South American fur seal	Aaus39	Rio Grande do Sul	Brazil	29°20’S, 49°’42’W	Yes
Arctocephalus australis	South American fur seal	Aaus40	Rio Grande do Sul	Brazil	29°20’S, 49°’42’W	No
Arctocephalus australis	South American fur seal	Aaus41	Rio Grande do Sul	Brazil	29°20’S, 49°’42’W	Yes
Arctocephalus australis	South American fur seal	Aaus42	Rio Grande do Sul	Brazil	29°20’S, 49°’42’W	Yes
Arctocephalus australis	South American fur seal	Aaus43	Falkland/Malvinas Is.	BOS*/Argentina	52°10’S, 60°55’W	No
Arctocephalus australis	South American fur seal	Aaus44	Falkland/Malvinas Is.	BOS*/Argentina	52°10’S, 60°55’W	Yes
Arctocephalus australis	South American fur seal	Aaus45	Falkland/Malvinas Is.	BOS*/Argentina	52°10’S, 60°55’W	Yes
Arctocephalus australis	South American fur seal	Aaus46	Falkland/Malvinas Is.	BOS*/Argentina	52°10’S, 60°55’W	Yes
Arctocephalus australis	South American fur seal	Aaus47	Falkland/Malvinas Is.	BOS*/Argentina	52°10’S, 60°55’W	No
Arctocephalus australis	South American fur seal	Aaus48	Falkland/Malvinas Is.	BOS*/Argentina	52°10’S, 60°55’W	Yes
Arctocephalus australis	South American fur seal	Aaus49	Falkland/Malvinas Is.	BOS*/Argentina	52°10’S, 60°55’W	Yes
Arctocephalus australis	South American fur seal	Aaus50	Falkland/Malvinas Is.	BOS*/Argentina	52°10’S, 60°55’W	Yes
Arctocephalus australis	South American fur seal	Aaus51	Falkland/Malvinas Is.	BOS*/Argentina	52°10’S, 60°55’W	Yes
Arctocephalus australis	South American fur seal	Aaus52	Falkland/Malvinas Is.	BOS*/Argentina	52°10’S, 60°55’W	Yes
Arctocephalus galapagoensis	Galapagos fur seal	Agal01	Cape Ibbetson	Ecuador	0°32’S, 90°43’W	No
Arctocephalus galapagoensis	Galapagos fur seal	Agal02	Cape Ibbetson	Ecuador	0°32’S, 90°43’W	No
Arctocephalus galapagoensis	Galapagos fur seal	Agal03	Punta Flores	Ecuador	0°05’S, 91°29’W	No
Arctocephalus galapagoensis	Galapagos fur seal	Agal04	Punta Flores	Ecuador	0°05’S, 91°29’W	No
Arctocephalus galapagoensis	Galapagos fur seal	Agal05	Punta Flores	Ecuador	0°05’S, 91°29’W	No
Arctocephalus galapagoensis	Galapagos fur seal	Agal06	Cape Marshall	Ecuador	0°01’N, 91°12’W	No
Arctocephalus galapagoensis	Galapagos fur seal	Agal07	Cape Marshall	Ecuador	0°01’N, 91°12’W	No
Arctocephalus galapagoensis	Galapagos fur seal	Agal08	Cape Marshall	Ecuador	0°01’N, 91°12’W	No
Arctocephalus galapagoensis	Galapagos fur seal	Agal09	Cape Marshall	Ecuador	0°01’N, 91°12’W	No
Arctocephalus galapagoensis	Galapagos fur seal	Agal10	Cape Marshall	Ecuador	0°01’N, 91°12’W	No
Arctocephalus forsteri	New Zealand fur seal	Afor01	Otago Harbour	New Zealand	45°49’S, 170°38E	No
Arctocephalus forsteri	New Zealand fur seal	Afor02	Otago Harbour	New Zealand	45°49’S, 170°38E	No
Arctocephalus forsteri	New Zealand fur seal	Afor03	Otago Harbour	New Zealand	45°49’S, 170°38E	No
Arctocephalus forsteri	New Zealand fur seal	Afor04	Otago Harbour	New Zealand	45°49’S, 170°38E	No
Arctocephalus forsteri	New Zealand fur seal	Afor05	Otago Harbour	New Zealand	45°49’S, 170°38E	No
Arctocephalus forsteri	New Zealand fur seal	Afor06	Otago Harbour	New Zealand	45°49’S, 170°38E	No
Arctocephalus forsteri	New Zealand fur seal	Afor07	Otago Harbour	New Zealand	45°49’S, 170°38E	No
Arctocephalus forsteri	New Zealand fur seal	Afor07	Otago Harbour	New Zealand	45°49’S, 170°38E	No
Arctocephalus forsteri	New Zealand fur seal	Afor09	Otago Harbour	New Zealand	45°49’S, 170°38E	No

*BOS = British Overseas Territory

Table S3.
Weighted pairwise F_{ST} from ddRAD-seq data. SAfs - South American fur seal: Brazil - BR, Argentina - AR, Falkland/Malvinas Islands - FK, and Chile - CH; Peruvian fur seal - Pfs; Galapagos fur seal - Gfs; and New Zealand fur seal - NZfs.

	SAfs BR	SAfs FK	SAfs CH	Pfs	Gfs	NZfs
**SAfs Argentina**	0.03	0.08	0.03	0.14	0.31	0.25
**SAfs Brazil**	-	0.09	0.05	0.14	0.32	0.24
**SAfs Falklands**	-	-	0.04	0.18	0.30	0.26
**SAfs Chile**	-	-	-	0.13	0.25	0.20
**Pfs**	-	-	-	-	0.22	0.29
**Gfs**	-	-	-	-	-	0.40

Table S4.
Origin of the mitochondrial DNA (matrilineal inheritance) of the Peruvian fur seal samples and the whole genomes studied here. For the WGS samples, the whole mitochondrial genome was obtained, and for the Pfs samples from the ddRAD-seq dataset, only the control region (CR). See Material and Methods for details. For more information on the samples, see table S2.

Sample	mtDNA	Locality
Aaus01	Peruvian fur seal	Punta San Juan
Aaus02	Peruvian fur seal	Punta San Juan
Aaus03	Peruvian fur seal	Punta San Juan
Aaus04	Peruvian fur seal	Punta San Juan
Aaus05	Peruvian fur seal	Punta San Juan
Aaus06	Peruvian fur seal	Punta San Juan
Aaus07	Peruvian fur seal	Punta San Juan
Aaus08	Peruvian fur seal	Punta San Juan
Aaus09 [a]	Peruvian fur seal	Punta San Juan
Aaus12	Peruvian fur seal	Punta San Juan
Aaus10	Peruvian fur seal	Isla Foca
Aaus11	Galapagos fur seal	Isla Foca
Aaus53	Galapagos fur seal	Isla Foca
Aaus19 [a]	South American fur seal	Chile
Aaus31	South American fur seal	Argentina
Aaus45 [a]	South American fur seal	Falkland Islands
Agal	Galapagos fur seal	Galapagos Islands
Afor	New Zealand fur seal	New Zealand

[a] Whole genome sequenced in this study.

Table S5.
F3-statistics for Peruvian fur seal (Pfs) population. f_3-statistics < 0 is evidence for genomic introgression from the Population combination column. Gfs (Galapagos fur seal), NZfs (New Zealand fur seal), and the different sampled localities of the South American fur seal (SAfs AR - Argentina, FK - Falkland Islands, CH - Chile). Bold lines highlight the significant signals of admixture with |Z-score| > 3.

Admixed Population	Population combination	f_3-statistics	Standard Error	Z-score
**Pfs**	**CH ; Gfs**	**-0.015**	**0.0003**	**-55.42**
**Pfs**	**AR ; Gfs**	**-0.015**	**0.0003**	**-55.27**
**Pfs**	**FK ; Gfs**	**-0.015**	**0.0003**	**-54.09**
**Pfs**	**Gfs ; NZfs**	**-0.007**	**0.0003**	**-27.28**
Pfs	AR ; NZfs	0.004	0.0003	13.43
Pfs	CH ; NZfs	0.004	0.0003	13.77
Pfs	FK ; NZfs	0.005	0.0003	17.17
Pfs	AR ; CH	0.013	0.0004	34.52
Pfs	AR ; FK	0.013	0.0004	35.08
Pfs	FK ; CH	0.013	0.0004	35.76

Table S6.
Relative and absolute values of the parameters estimated by BP&P with MSCi (Multispecies Coalescent model in the presence of introgression). U, V, X, S, H, R, and T represent the *tau* values in the events of the species network described in the table's footnote.

	Relative values				Absolute age		
	Median value	Percentile			Median age	Percentil	
tau		ESS	5%	95%		5%	95%
U	3.54E-04	1,738	3.15E-04	3.90E-04	1,200,000	1,200,000	1,200,000
V	1.90E-04	1,746	1.82E-04	1.98E-04	644,877	693,333	609,231
X	1.90E-04	1,746	1.82E-04	1.98E-04	644,877	693,333	609,231
S	1.27E-04	1,754	1.20E-04	1.31E-04	428,450	457,143	403,077
H	1.27E-04	1,754	1.20E-04	1.31E-04	428,450	457,143	403,077
R	1.90E-04	1,746	1.82E-04	1.98E-04	644,877	693,333	609,231
T	1.27E-04	1,754	1.20E-04	1.31E-04	428,450	457,143	403,077
**Introgression estimation**							
phi_X	33.00%	1,127	27.00%	38.00%			
phi_H	2.41%	3,845	0.00%	7.00%			

((((A, (B)H[&phi=0.5,&tau-parent=no])S)X[&phi=0.5,&tau-parent=no], D)V, ((H[&tau-parent=no], C)T ,X)R)U;
A: South American fur seal; B: Peruvian fur seal; C: Galapagos fur seal; and D: New Zealand fur seal.

REFERENCES

1. J. Mallet, Hybrid speciation. *Nature* **446**, 279–283 (2007).

2. J. Mallet, Hybridization as an invasion of the genome. *Trends Ecol. Evol.* **20**, 229–237 (2005).

3. R. Abbott, D. Albach, S. Ansell, J. W. Arntzen, S. J. E. Baird, N. Bierne, J. Boughman, A. Brelsford, C. A. Buerkle, R. Buggs, R. K. Butlin, U. Dieckmann, F. Eroukhmanoff, A. Grill, S. H. Cahan, J. S. Hermansen, G. Hewitt, A. G. Hudson, C. Jiggins, J. Jones, B. Keller, T. Marczewski, J. Mallet, P. Martinez-Rodriguez, M. Möst, S. Mullen, R. Nichols, A. W. Nolte, C. Parisod, K. Pfennig, A. M. Rice, M. G. Ritchie, B. Seifert, C. M. Smadja, R. Stelkens, J. M. Szymura, R. Väinölä, J. B. W. Wolf, D. Zinner, Hybridization and speciation. *J. Evol. Biol.* **26**, 229–246 (2013).

4. A. Runemark, C. N. Trier, F. Eroukhmanoff, J. S. Hermansen, M. Matschiner, T. O. Elgvin, G. P. Sætre, Variation and constraints in hybrid genome formation. *Nat. Ecol. Evol.* **2**, 549–556 (2018).

5. A. Runemark, M. Vallejo-Martin, J. I. Meier, Eukaryote hybrid genomes. *PLOS Genet.* **15**, e1008404 (2019).

6. R. J. Abbott, N. H. Barton, J. M. Good, Genomics of hybridization and its evolutionary consequences. *Mol. Ecol.* **25**, 2325–2332 (2016).

7. D. A. Marques, J. Meier, O. Seehausen, A combinatorial view on speciation and adaptive radiation. *Trends Ecol. Evol.* **34**, 531–544 (2019).

8. A. W. Nolte, D. Tautz, Understanding the onset of hybrid speciation. *Trends Genet.* **26**, 54–58 (2010).

9. N. B. Edelman, J. Mallet, Prevalence and adaptive impact of introgression. *Annu. Rev. Genet.* **55**, 265–283 (2021).

10. G. Nieto Feliner, I. Álvarez, J. Fuertes-Aguilar, M. Heuertz, I. Marques, F. Moharrek, R. Piñeiro, R. Riina, J. A. Rosselló, P. S. Soltis, I. Villa-Machío, Is homoploid hybrid speciation that rare? An empiricist's view. *Heredity* **118**, 513–516 (2017).

11. M. Schumer, G. G. Rosenthal, P. Andolfatto, How common is homoploid hybrid speciation? *Evolution* **68**, 1553–1560 (2014).

12. Z. Wang, M. Kang, J. Li, Z. Zhang, Y. Wang, C. Chen, Y. Yang, J. Liu, Genomic evidence for homoploid hybrid speciation between ancestors of two different genera. *Nat. Commun.* **13**, 1987 (2022).

13. D. Bolnick, A. K. Hund, P. Nosil, F. Peng, M. Ravinet, S. Stankowski, S. Subramanian, J. B. W. Wolf, R. Yukilevich, A multivariate view of the speciation continuum. *Evolution* **1**:318–328 (2023).

14. Y. Sun, Z. Lu, X. Zhu, H. Ma, Genomic basis of homoploid hybrid speciation within chestnut trees. *Nat. Commun.* **11**, 3375 (2020).

15. D. Ru, Y. Sun, D. Wang, Y. Chen, T. Wang, Q. Hu, R. J. Abbott, J. Liu, Population genomic analysis reveals that homoploid hybrid speciation can be a lengthy process. *Mol. Ecol.* **27**, 4875–4887 (2018).

16. S. A. Taylor, E. L. Larson, Insights from genomes into the evolutionary importance and prevalence of hybridization in nature. *Nat. Ecol. Evol.* **3**, 170–177 (2019).

17. R. Adavoudi, M. Pilot, Consequences of hybridization in mammals: A systematic review. *Genes* **13**, 50 (2022).

18. F. Lopes, L. R. Oliveira, A. Kessler, Y. Beux, E. Crespo, S. Cárdenas-Alayza, P. Majluf, M. Sepúlveda, R. L. Brownell Jr., V. Franco-Trecu, D. Páez-Rosas, J. Chaves, C. Loch, B. C. Robertson, K. Acevedo-Whitehouse, F. R. Elorriaga-Verplancken, S. P. Kirkman, C. R. Peart, J. B. W. Wolf, S. L. Bonatto, Phylogenomic discordance in the eared seals is best explained by incomplete lineage sorting following explosive radiation in the Southern Hemisphere. *Syst. Biol.* **70**, 786–802 (2021).

19. F. Trillmich, *Arctocephalus galapagoensis*. *The IUCN Red List of Threatened Species*. 2015:e.T2057A45223722 (2015); https://dx.doi.org/10.2305/IUCN.UK.2015-2.RLTS.T2057A45223722.en.

20. B. L. Chilvers, S. D. Goldsworthy, *Arctocephalus forsteri*. *The IUCN Red List of Threatened Species*. 2015. e.T41664A45230026 (2015); https://dx.doi.org/10.2305/IUCN.UK.2015-2.RLTS.T41664A45230026.en.

21. S. Cárdenas-Alayza, L. Oliveira, E. Crespo, *Arctocephalus australis*. *The IUCN Red List of Threatened Species*. e.T2055A45223529 (2016).

22. L. Oliveira, E. Hingst-Zaher, J. S. Morgante, Size and shape sexual dimorphism in the skull of the South American fur seal, *Arctocephalus australis* (Zimmermann, 1783) (Carnivora: Otariidae). *Lat. Am. J. Aquat. Mamm.* **4**, 27–40 (2005).

23. L. R. de Oliveira, J. I. Hoffman, E. Hingst-Zaher, P. Majluf, M. M. C. Muelbert, J. S. Morgante, W. Amos, Morphological and genetic evidence for two evolutionarily significant units (ESUs) in the South American fur seal, *Arctocephalus gazella*, *Conserv. Genet.* **9**, 1451–1466 (2008).

24. J. I. Túnez, H. L. Cappozzo, H. Paves, D. A. Albareda, M. H. Cassini, The role of Pleistocene glaciations in shaping the genetic structure of South American fur seals (*Arctocephalus australis*). *N. Z. J. Mar. Freshw. Res.* **47**, 139–152 (2013).

25. H. J. Pavés, R. P. Schlatter, V. Franco-Trecu, E. Paez, W. Siefeld, V. Araos, R. Giesecke, L. M. Batallés, H. L. Capozzo, Breeding season of the South American fur seal (*Arctocephalus australis*, Otariidae: Carnivora): New data for establishing independent evolutionary histories? *Rev. Biol. Mar. Oceanogr.* **51**, 241–253 (2016).

26. A. Berta, M. Churchill, Pinniped taxonomy: Review of currently recognized species and subspecies, and evidence used for their description. *Mamm. Rev.* **42**, 207–234 (2012).

27. L. R. de Oliveira, R. L. Brownell Jr., Taxonomic status of two subspecies of South American fur seals: *Arctocephalus australis australis* vs. *A. a. gracilis*. *Mar. Mamm. Sci.* **30**, 1258–1263 (2014).

28. R. Aguilar-Arakaki, Población del lobo fino *Arctocephalus australis* en la costa peruana en el periodo 2016-2019. *Bol. Inst. Mar. Perú* **36**, 188–204 (2021).

29. B. J. Evans, N. S. Upham, G. B. Golding, R. A. Ojeda, A. A. Ojeda, Evolution of the largest mammalian genome. *Genome Biol. Evol.* **9**, 1711–1724 (2017).

30. A. Beilts, I. M. Rahn, M. C. G. Moreno, J. Loureiro, M. S. Merani, Mitotic and meiotic analysis in *Arctocephalus australis* (Otariidae). *Hereditas* **131**, 33–37 (1999).

31. T. O. Elgvin, C. N. Trier, O. K. Tørresen, I. J. Hagen, S. Lien, A. J. Nederbragt, M. Ravinet, H. Jensen, G.-P. Sætre, The genomic mosaicism of hybrid speciation. *Sci. Adv.* **3**, e1602996 (2017).

32. P. Rodrigues, M. Seguel, J. Gutiérrez, H. Pavés, C. Verdugo, Genetic connectivity of the South American fur seal (*Arctocephalus australis*) across Atlantic and Pacific oceans revealed by mitochondrial genes. *Aquat. Conserv. Mar. Freshw. Ecosyst.* **28**, 315–323 (2018).

33. T. Yonezawa, N. Kohno, M. Hasegawa, The monophyletic origin of sea lions and fur seals (Carnivora; Otariidae) in the Southern Hemisphere. *Gene* **441**, 89–99 (2009).

34. N. Patterson, P. Moorjani, Y. Luo, S. Mallick, N. Rohland, Y. Zhan, T. Genschoreck, T. Webster, D. Reich, Ancient admixture in human history. *Genetics* **192**, 1065–1093 (2012).

35. M. Malinsky, M. Matschine, H. Svardal, Dsuite - Fast D-statistics and related admixture evidence from VCF files. *Mol. Ecol. Res.* **21**, 584–595 (2021).

36. P. D. Blischak, J. Chifman, A. D. Wolfe, L. S. Kubatko, HyDe: A Python package for genome-scale hybridization detection. *Syst. Biol.* **67**, 821–829 (2018).

37. S. Kong, L. S. Kubatko, Comparative performance of popular methods for hybrid detection using genomic data. *Syst. Biol.* **70**, 891–907 (2021).

38. J. K. Pickrell, J. K. Pritchard, Inference of population splits and mixtures from genome-wide allele frequency data. *PLOS Genet.* **8**, e1002967 (2012).

39. S. M. Boca, L. Huang, N. A. Rosenberg, On the heterozygosity of an admixed population. *J. Math. Biol.* **81**, 1217–1250 (2020).

40. P. Majluf, F. Trillmich, Distribution and abundance of sea lions (*Otaria byronia*) and fur seals (*Arctocephalus australis*) in Peru. *Zeitschrift für Säugetierkunde* **46**, 384–393 (1981).

41. A. G. Fabritzek, E. M. Griebeler, J. W. Kadereit, Hybridization, ecogeographical displacement and the emergence of new lineages–A genotyping-by-sequencing and ecological niche and species distribution modelling study of *Sempervivum tectorum* L. (Houseleek). *J. Evol. Biol.* **34**, 830–844 (2021).

42. T. D. Price, M. M. Bouvier, The evolution of F_1 postzygotic incompatibilities in birds. *Evolution* **56**, 2083–2089 (2002).

43. M. L. Lancaster, N. J. Gemmell, S. Negro, S. Goldsworthy, P. Sunnucks, Ménage à trois on Macquarie Island: Hybridization among three species of fur seal (*Arctocephalus* spp.) following historical population extinction. *Mol. Ecol.* **15**, 3681–3692 (2006).

44. M. N. Schaurich, F. Lopes, L. R. Oliveira, Hybridization phenomenon in cetacean and pinniped species. *Neotrop. Biol. Conserv.* **7**, 199–209 (2012).

45. D. Páez-Rosas, J. Torres, E. Espinoza, A. Marchetti, H. Seim, M. Riofrío-Lazo, Declines and recovery in endangered Galapagos pinnipeds during the El Niño event. *Sci. Rep.* **11**, 8785 (2021).

46. L. R. de Oliveira, D. Meyer, J. Hoffman, P. Majluf, J. S. Morgante, Evidence of a genetic bottleneck in an El Niño affected population of South American fur seals, *Arctocephalus australis*. *J. Mar. Biol. Assoc. U.K.* **89**, 1717–1725 (2009).

47. S. Cárdenas-Alayza, L. Oliveira, *Arctocephalus australis* (Peruvian/Northern Chilean subpopulation. *The IUCN Red List of Threatened Species*. e.T72050476A72050985 (2016).

48. G. T. Rustic, P. J. Polissar, A. C. Ravelo, S. M. White, Modulation of late Pleistocene ENSO strength by the tropical Pacific thermocline. *Nat. Commun.* **11**, 5377 (2020).

49. L. Milmann, R. Machado, L. R. de Oliveira, P. H. Ott, Far away from home: Presence of fur seal (*Arctocephalus* sp.) in the equatorial Atlantic Ocean. *Polar Biol.* **42**, 817–822 (2019).

50. D. Páez-Rosas, D. Pazmiño, M. Riofrío-Lazo, Unprecedented records of Guadalupe and Juan Fernández fur seals in the Galapagos archipelago. *Aquat. Mamm.* **46**, 549–555 (2020).

51. D. Páez-Rosas, L. A. Valdovinos, F. R. Elorriaga-Verplancken, Northernmost record of the Galapagos fur seal (*Arctocephalus galapagoensis*): A consequence of anomalous warm conditions around the Galapagos Archipelago. *Aquat. Mamm.* **43**, 629–634 (2017).

52. E. S. Poloczanska, M. T. Burrows, C. J. Brown, J. García Molinos, B. S. Halpern, O. Hoegh-Guldberg, C. V. Kappel, P. J. Moore, A. J. Richardson, D. S. Schoeman, W. J. Sydeman, Responses of marine organisms to climate change across oceans. *Front. Mar. Sci.* **3**, 62 (2016).

53. J. E. King, The otariid seals of the Pacific coast of America in *Bull British Mus (*Natural History, 1954), vol. 2, pp. 309–337.

54. J. E. King, *Seals of the world* (Cornell University Press, Ithaca, NY, ed. 2nd, 1983).

55. E. Humble, K. K. Dasmahapatra, A. Martinez-Barrio, I. Gregório, J. Forcada, A.-C. Polikeit, S. D. Goldsworthy, M. E. Goebel, J. Kalinowski, J. B. W. Wolf, J. I. Hoffman, RAD sequencing and a hybrid Antarctic fur seal genome assembly reveal rapidly decaying linkage disequilibrium, global population structure and evidence for inbreeding. *G3 (Bethesda)* **8**, 2709–2722 (2018).

56. T. Kess, J. Gross, F. Harper, E. G. Boulding, Low-cost ddRAD method of SNP discovery and genotyping applied to the periwinkle *Littorina saxatilis*. *J. Mollus. Stud.* **82**, 104–109 (2016).

57. B. K. Peterson, J. N. Weber, E. H. Kay, H. S. Fisher, H. E. Hoekstra, Double digest RADseq: An inexpensive method for de novo SNP discovery and genotyping in model and non-model species. *PLOS ONE* **7**, e37135 (2012).

58. J. M. DaCosta, M. D. Sorenson, Amplification biases and consistent recovery of loci in a double-digest RAD-seq protocol. *PLOS ONE* **9**, e106713 (2014).

59. A. D. Foote, Y. Liu, G. W. C. Thomas, T. Vinař, J. Alföldi, J. Deng, S. Dugan, C. E. van Elk, M. E. Hunter, V. Joshi, Z. Khan, C. Kovar, S. L. Lee, K. Lindblad-Toh, A. Mancia, R. Nielsen, X. Qin, J. Qu, B. J. Raney, N. Vijay, J. B. W. Wolf, M. W. Hahn, D. M. Muzny, K. C. Worley, M. T. P. Gilbert, R. A. Gibbs, Convergent evolution of the genomes of marine mammals. *Nat. Genet.* **47**, 272–275 (2015).

60. M. Schubert, L. Ermini, C. D. Sarkissian, H. Jónsson, A. Ginolhac, R. Schaefer, M. D. Martin, R. Fernández, M. Kircher, M. McCue, E. Willerslev, L. Orlando, Characterization of ancient and modern genomes by SNP detection and phylogenomic and metagenomic analysis using PALEOMIX. *Nat. Protoc.* **9**, 1056–1082 (2014).

61. M. Schubert, S. Lindgreen, L. Orlando, AdapterRemoval v2: Rapid adapter trimming, identification, and read merging. *BMC Res. Notes* **9**, 88 (2016).

62. H. Li, R. Durbin, Fast and accurate short read alignment with Burrows-Wheeler transform. *Bioinformatics* **25**, 1754–1760 (2009).

63. B. Arnold, R. B. Corbett-Detig, D. Hartl, K. Bomblies, RADseq underestimates diversity and introduces genealogical biases due to nonrandom haplotype sampling. *Mol. Ecol.* **22**, 3179–3190 (2013).

64. M. Gautier, K. Gharbi, T. Cezard, J. Foucaud, C. Kerdelhué, P. Pudlo, J.-M. Cornuet, A. Estoup, The effect of RAD allele dropout on the estimation of genetic variation within and between populations. *Mol. Ecol.* **22**, 3165–3178 (2013).

65. A. McKenna, M. Hanna, E. Banks, A. Sivachenko, K. Cibulskis, A. Kernytsky, K. Garimella, D. Altshuler, S. Gabriel, M. Daly, M. A. DePristo, The Genome Analysis Toolkit: A MapReference framework for analyzing next-generation DNA sequencing data. *Genome Res.* **20**, 1297–1303 (2010).

66. T. S. Korneliussen, A. Albrechtsen, R. Nielsen, ANGSD: Analysis of Next Generation Sequencing Data. *BMC Bioinformatics* **15**, 356 (2014).

67. B. A. S. de Medeiros, B. D. Farrell, Whole-genome amplification in double-digest RADseq results in adequate libraries but fewer sequenced loci. *PeerJ.* **6**, e5089 (2018).

68. R. A. C. Dos Santos, G. H. Goldman, D. M. Riaño-Pachón, ploidyNGS: Visually exploring ploidy with Next Generation Sequencing data. *Bioinformatics* **33**, 2575–2576 (2017).

69. P. Danecek, A. Auton, G. Abecasis, C. A. Albers, E. Banks, M. A. DePristo, R. E. Handsaker, G. Lunter, G. T. Marth, S. T. Sherry, G. McVean, R. Durbin; 1000 Genomes Project Analysis Group, The variant call format and VCFtools. *Bioinformatics* **27**, 2156–2158 (2011).

70. N. Singh, J. Raupp, D.-H. Koo, B. Friebe, B. Gill, J. Poland, In-silico detection of aneuploidy and chromosomal deletions in wheat using genotyping-by-sequencing. *Plant Methods* **16**, 45 (2020).

71. K. Okonechnikov, A. Conesa, F. García-Alcalde, Qualimap 2: Advanced multi-sample quality control for high-throughput sequencing data. *Bioinformatics* **32**, 292–294 (2016).

72. A. R. Quinlan, I. M. Hall, BEDTools: A flexible suite of utilities for comparing genomic features. *Bioinformatics* **26**, 841–842 (2010).

73. J. Meisner, A. Albretchtsen, Inferring population structure and admixture proportions in low-depth NGS data. *Genetics* **210**, 719–731 (2018).

74. R. M. Francis, Pophelper: An R package and web app to analyse and visualize population structure. *Mol. Ecol. Resour.* **17**, 27–32 (2017).

75. H. A. Ogilvie, R. R. Bouckaert, A. J. Drummond, StarBEAST2 Brings faster species tree inference and accurate estimates of substitution rates. *Mol. Biol. Evol.* **34**, 2101–2114 (2017).

76. C. Zhang, M. Rabiee, E. Sayyari, S. Mirarab, ASTRAL-III: Polynomial time species tree reconstruction from partially resolved gene trees. *BMC Bioinformatics* **19**, 153 (2018).

77. S. Capella-Gutiérrez, J. M. Silla-Martínez, T. Gabaldón, trimAl: A tool for automated alignment trimming in large-scale phylogenetic analyses. *Bioinformatics* **25**, 1972–1973 (2009).

78. A. Stamatakis, RAxML version 8: A tool for phylogenetic analysis and post-analysis of large phylogenies. *Bioinformatics* **30**, 1312–1313 (2014).

79. D. Darriba, G. L. Taboada, R. Doallo, D. Posada, jModelTest 2: More models, new heuristics and parallel computing. *Nat. Methods* **9**, 772 (2012).

80. S. Kumar, G. Stecher, M. Li, C. Knyaz, K. Kumar, Mega X: Molecular Evolutionary Genetics Analysis across computing platforms. *Mol. Biol. Evol.* **35**, 1547–1549 (2018).

81. F. Lopes, J. I. Hoffman, V. H. Valiati, S. L. Bonatto, J. B. W. Wolf, F. Trillmich, L. R. Oliveira, Fine-scale matrilineal population structure in the Galapagos fur seal and its implications for conservation management. *Conserv. Genet.* **16**, 1099–1113 (2015).

82. L. P. Wynen, S. D. Goldsworthy, S. J. Insley, M. Adams, J. W. Bickham, J. Francis, J. P. Gallo, A. R. Hoelzel, P. Majluf, R. W. G. White, R. Slade, Phylogenetic relationships within the eared seals (Otariidae: Carnivora): Implications for the historical biogeography of the family. *Mol. Phylogenet. Evol.* **21**, 270–284 (2001).

83. S. H. Martin, S. M. Van Belleghem, Exploring evolutionary relationships across the genome using topology weighting. *Genetics* **206**, 429–438 (2017).

84. A. M. Harris, M. De Giorgio, Admixture and ancestry inference from ancient and modern samples through measures of population genetic drift. *Hum. Biol.* **89**, 21–46 (2017).

85. B. M. Peter, Admixture, population structure, and F-statistics. *Genetics* **202**, 1485–1501 (2016).

86. Y. Zhenge, A. Janke, Gene flow analysis method, the D-statistic, is robust in a wide parameter space. *BMC Bioinformatics* **19**, 10 (2018).

87. K. Leppälä, S. V. Nielsen, T. Mailund, admixturegraph: An R package for admixture graph manipulation and fitting. *Bioinformatics* **33**, 1738–1740 (2017).

88. M. Malinsky, H. Svardal, A. M. Tyers, E. A. Miska, M. J. Genner, G. F. Turner, R. Durbin, Whole-genome sequences of Malawi cichlids reveal multiple radiations interconnected by gene flow. *Nat. Ecol. Evol.* **2**, 1940–1955 (2018).

89. E. Y. Durand, N. Patterson, D. Reich, M. Slatkin, Testing for ancient admixture between closely related populations. *Mol. Biol. Evol.* **28**, 2239–2252 (2011).

90. D. Reich, R. E. Green, M. Kircher, J. Krause, N. Patterson, E. Y. Durand, B. Viola, A. W. Briggs, U. Stenzel, P. L. Johnson, T. Maricic, J. M. Good, T. Marques-Bonet, C. Alkan, Q. Fu, S. Mallick, H. Li, M. Meyer, E. E. Eichler, M. Stoneking, M. Richards, S. Talamo, M. V. Shunkov, A. P. Derevianko, J.-J. Hublin, J. Kelso, M. Slatkin, S. Pääbo, Genetic history of an archaic hominin group from Denisova Cave in Siberia. *Nature* **468**, 1053–1060 (2010).

91. D. Wen, Y. Yu, J. Zhu, L. Nakhleh, Inferring phylogenetic networks using PhyloNet. *Syst. Biol.* **67**, 735–740 (2018).

92. H. M. Lam, O. Ratmann, M. F. Boni, Improved algorithmic complexity for the 3SEQ recombination detection algorithm. *Mol. Biol. Evol.* **35**, 247–251 (2018).

93. W. R. Rice, Analyzing tables of statistical tests. *Evolution* **43**, 223–225 (1989).

94. T. Flouri, X. Jiao, B. Rannala, Z. Yang, A Bayesian implementation of the multispecies coalescent model with introgression for phylogenomic analysis. *Mol. Biol. Evol.* **37**, 1211–1223 (2020).

95. B. Rannala, Z. Yang, Bayes estimation of species divergence times and ancestral population sizes using DNA sequences from multiple loci. *Genetics* **164**, 1645–1656 (2003).

96. J.-M. Cornuet, P. Pudlo, J. Veyssier, A. Dehne-Garcia, M. Gautier, R. Leblois, J.-M. Marin, A. Estoup, DIYABC v2.0: A software to make approximate Bayesian computation inferences about

population history using single nucleotide polymorphism, DNA sequence and microsatellite data. *Bioinformatics* **30**, 1187–1189 (2014).

97. C. C. Chang, C. C. Chow, L. C. Tellier, S. Vattikuti, S. M. Purcell, J. J. Lee, Second-generation PLINK: Rising to the challenge of larger and richer datasets. *GigaScience* **4**, 7 (2015).
